# ALD-Grown ZnO TFTs Patterned by High-Resolution Reverse-Offset Printing

**DOI:** 10.1021/acsami.5c03321

**Published:** 2025-06-03

**Authors:** Fei Liu, Asko Sneck, Patrik Eskelinen, Olli Halonen, Liam Gillan, Jaakko Leppäniemi

**Affiliations:** 3259 VTT Technical Research Centre of Finland Ltd, Espoo 02150, Finland

**Keywords:** thin-film transistor, atomic layer deposition, zinc oxide, high-resolution printing, reverse-offset printing, printed electronics

## Abstract

Zinc oxide (ZnO) is a benign and earth-abundant semiconductor material that has been applied in thin-film transistors (TFTs) for decades and can be used in biodegradable, transient, and biocompatible devices. Printing as an alternative fabrication method to conventional TFT manufacturing methods can deliver some benefits, such as simultaneous film deposition and patterning, good scalability, low cost, and material-saving features. However, the high annealing temperature needed for ink-to-metal oxide conversion and film densification, compounded by the poor patterning resolution of conventional printing methods, still limits the use of printing in the fabrication of flexible metal oxide TFTs. Atomic layer deposition (ALD) has recently emerged as a promising fabrication method for high-performance metal oxide TFTs that can offer more conformal film growth, precise film thickness, and higher film quality at low temperatures compared to sputtering, spin coating, or printing. Although ALD-based ZnO TFTs patterned with photolithography exhibit good electrical properties, they cannot be readily scaled to a high-throughput fabrication. Very little attention has been paid so far to the combination of low-temperature ALD growth with printing to obtain more scalable manufacturing of high-performance thin-film electronics. To overcome this challenge, we propose high-resolution reverse-offset printing (ROP) of a simple polymer resist to pattern an ALD-grown ZnO film at few μm resolution to fabricate TFTs. In this work, we report high-performance ZnO TFTs that are ALD-grown at a low temperature of 150 °C and ROP-patterned with promising stability and uniformity, a high field-effect mobility (μ_FE_) of ∼16.6 cm^2^ (Vs)^−1^, an almost zero turn-on voltage (*V*_on_) of ∼−0.49 V, a high current on–off ratio (*I*_on_/*I*_off_) of >10^8^, a low operation voltage (*V*_op_) of ≤5 V, and a negligible hysteresis (*V*_hyst_) of ∼0.13 V. The combination of ALD and the ROP-patterning process could be developed further to fabricate fully flexible high-resolution metal oxide TFT-based circuits in the future.

## Graphical abstract

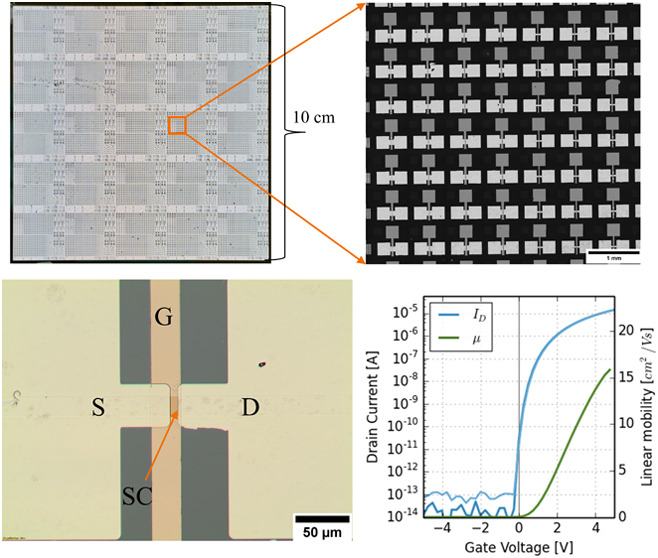

## Introduction

Oxide thin-film transistors (TFTs) are key components in displays and flat-panel detectors. They have been an active research topic for decades due to their transparency,^1^ flexibility,^2^ high charge carrier mobility,^3^ and potential for scalable processes like printing.^4^ Among them, the zinc oxide (ZnO) semiconductor material has been widely employed in TFTs because of its straightforward composition and low cost.^5^ Compared to other oxide TFTs, such as those based on indium oxide (In_2_O_3_) and indium gallium zinc oxide (IGZO), ZnO-based TFTs employ more benign and earth-abundant materials and offer the potential to be used in nontoxic, transient, and biocompatible devices.^6^ This is particularly important in applications where recyclability or biodegradability of the electronics is critical, such as biodegradable sensors in precision agriculture^7^ or improving the sustainability of wearable electronic systems with material or component recovery.^8^ Besides the higher abundance and annual production of Zn over In, Zn can even act as a micronutrient to plant growth, thus providing a relatively safe disposal route in biodegradable devices.^9,10^ Additionally, other potential application areas such as implantable electronics and on-skin sensor patches are also demanding this kind of benign and biocompatible material.^11,12^ Regarding the preparation of the ZnO-based semiconductor layer, several fabrication methods have been reported using vacuum processes such as sputtering^13^ or atomic layer deposition (ALD)^14^ and using solution processes such as spray pyrolysis,^15^ spin coating,^16^ and printing.^17^ Recent publications on these different deposition and patterning methods of ZnO-based TFTs are summarized in Table S1. Out of these, the ALD-grown ZnO TFTs patterned by photolithography (PL) offer the highest field-effect mobility, excellent thickness uniformity, and control for film stoichiometry at relatively low deposition temperatures.^18^ On the other hand, printing is considered a scalable, low-cost, and material-saving fabrication method.^17^ But the low quality, density, and purity of printed metal oxide semiconductor thin films, the requirement for a high annealing temperature after printing, the low electrical performance of the semiconductor layer, and the large device size due to the coarse patterning resolution and typical overlay alignment of conventional printing methods (>10 μm) still limit the usability of printing in the fabrication of high-performance ZnO TFTs.

The estimated benefits of different fabrication methods of TFTs including printing and patterning of vacuum-deposited layers are summarized in Table S2. Notably, a method where all layers of the TFTs, i.e., gate (G), gate insulator (GI), semiconductor (SC), and source/drain (S/D), could be patterned would be beneficial for process integration. When compared to metals that can be patterned with PL, the material palette of printable metals is very limited (mostly Ag, Cu, and Au) and those are typically nonoptimal as S/D electrodes to n-type oxide semiconductors due to the large contact resistance, poor migration resistance, and stability problems.^19^ Therefore, fabrication methods that provide high-resolution patterning, good scalability, reproducible multilayer patterning, low-temperature processing, and a wide material palette, including materials that provide low contact resistance as well as high-mobility oxide semiconductors, show promise for the scalable fabrication of high-performance flexible TFTs.

So far, little attention has been paid to investigating the potential benefits of combining low-temperature ALD and scalable printing for the manufacturing of ZnO-based TFTs. To the best of our knowledge, only two other groups have previously studied combining an ALD-grown semiconductor with print patterning. In 2013, Levy et al. demonstrated ALD-based ZnO TFTs that were patterned by inkjet printing of an inhibitor layer for area-selective ALD, but the resolution of these devices with a channel width (*W*) of 400 μm and a channel length (*L*) of 100 μm was low for practical circuit implementation.^20^ In 2020, Cho et al. combined ALD and electrohydrodynamic (EHD) jet printing to fabricate tin-doped zinc oxide (ZTO) TFTs with device dimensions down to *L* = 5 μm. However, the low on/off current ratio (*I*_on_/*I*_off_) (∼10^5^), the large operation voltage (*V*_op_) (≤50 V), and the need for a high postdeposition annealing temperature of 500 °C limit the applicability of such devices.^21^ Recently, high-resolution reverse-offset printing (ROP) has gained interest in the fabrication of thin-film devices including TFTs.^22^ The ROP of a simple polymer resist has been reported to pattern vacuum-deposited (evaporated or sputtered) metal or metal oxide layers at μm resolution without the need for a high-temperature annealing step using the lift-off (LO) process.^23,24^ A large area reproducibility and high uniformity with 4–6% parameter variations in thickness, sheet resistance, and resistivity were reported for a large area in ROP LO metals with >99% yield.^25^ In contrast to lithography, ROP is better suited for scalable high-throughput, even roll-to-roll (R2R), manufacturing processes.^26,27^

In this paper, we successfully fabricated ALD-grown ZnO TFTs by patterning the metal G, S/D, and the ZnO SC by a high-resolution ROP polymer resist layer. Figure 1 presents the different state of art in terms of the process temperature (*T*), *L*, and mobility (μ) of ZnO-based TFTs with different deposition and patterning methods. All of the references presenting results with a low-temperature (*T* <150 °C) process are marked with red color. Compared to the other ZnO TFTs shown in Figure 1 and Table S1, our devices with ALD-grown ZnO patterned with ROP can be fabricated at relatively low temperatures (150 °C) with promising stability and uniformity, a high field-effect electron mobility (μ_FE_) of ∼16.6 cm^2^ (Vs)^−1^, a nearly zero turn-on voltage (*V*_on_) of ∼−0.49 V, a high *I*_on_/*I*_off_ ratio of >10^8^, a low *V*_op_ of ≤5 V, and a negligible hysteresis (*V*_hyst_) of ∼0.13 V. Additionally, the size of TFTs is scaled down to *W* = 20 μm and *L* = 7.5 μm while still exhibiting a relatively high μ_FE_ of ∼15.1 cm^2^ (Vs)^−1^, a low *V*_op_, and the same order of *I*_on_/*I*_off_ (>10^8^) as the larger TFTs. Notably, our results show the smallest channel length attained for ZnO TFTs with printing-based patterning. In addition, only one report shown in Figure 1 features ALD-grown ZnO TFTs that are fabricated at 100 °C and exhibit a higher μ ∼ 45.3 cm^2^ (Vs)^−1^ and a smaller *L* of 3 μm than our results but feature patterning by standard PL.^28^ These indicate that our combination of high-resolution print patterning and ALD deposition shows good promise for scalable low-temperature fabrication of TFTs at a similar level as PL-patterned TFTs. The presented batch-based process is compatible with existing semiconductor lines since it is using standard vacuum deposition tools, and the role of the mask aligner is replaced with ROP. However, we believe that the combination of low-temperature ALD and ROP patterning can be further developed for scalable R2R manufacturing of metal-oxide-based flexible electronics. Moreover, the demonstrated 150 °C processing temperature could be pushed even lower to allow utilization of the proposed process for the fabrication of high-performance circuits on transient/biodegradable substrates. Such biodegradable circuits would help to increase the complexity of Si-free systems and avoid the integration of Si-based chips that are energy-intensive to manufacture, e.g., in single-use wearable sensors or precision agriculture.

**Figure 1:**
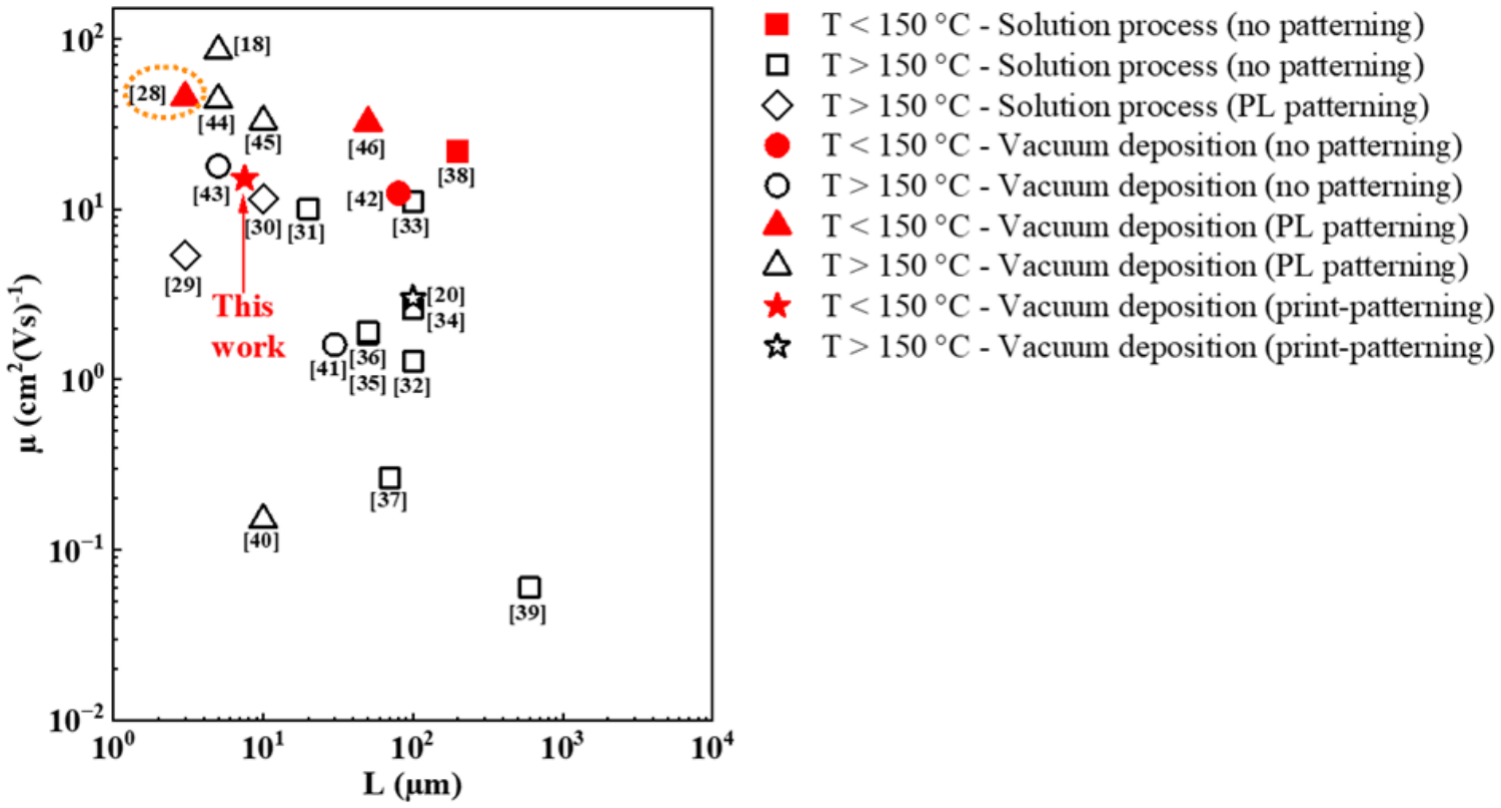
Comparison of state of art in terms of the process temperature (*T*), channel length (*L*), and mobility (μ) of ZnO-based TFTs with different deposition and patterning methods based on Table S1. The corresponding references inside are refs 18,20,28–46 .

## Experimental Section

### Patterning of TFTs with ROP

Patterning of TFT layers was conducted by the LO and etching processes using ROP of a single polymer resist layer as a patterning mask.^23^ In this study, a 3.5 wt % hygroscopic polymer poly­(4-vinylphenol) (PVPh) (Polysciences, MW ∼ 22,000, polydispersity ∼ 5) was dissolved in a 1:3 ratio of ethyl lactate:ethyl acetate solution with 0.2 wt % BYK-355 surfactant. Ethyl acetate was selected as the main solvent because of its low surface tension property. The ratio of two solvents was tailored and selected after investigating the film-forming capability and the drying conditions of the polymer resist on a polydimethylsiloxane (PDMS) printing blanket. The addition of ethyl lactate (29.2 mN/m, 154 °C, 2 mm Hg (20 °C)) with a higher boiling point and lower vapor pressure than ethyl acetate (24 mN/m, 77.1 °C, 73 mm Hg (20 °C)) can slow down the drying process of the polymer ink. In addition, the surfactant was added to improve leveling and reduce the formation of pin holes in the polymer resist film. The viscosity of the ink was <5 mPa and the surface tension was ∼24 mN m^–1^. The final polymer resist layer patterned with μm-level resolution can be used either as a sacrificial mask in the LO process or as a protective layer against the etchant during patterning processes. Therefore, it is possible to pattern all of the necessary TFT layers using a single ROP ink. The residual material from the poly­(4-vinylphenol) (PVPh) resist could have some impact on the TFT device performance, such as presenting an obstacle for charge transfer across the interface between the ZnO semiconductor and S/D contacts. The atomic force microscopy (AFM) measurements presented in Figure S1 revealed a similar surface morphology, a surface root-mean-square roughness (*R*_q_), and an average roughness (*R*_a_) for both ZnO with no PVPh (*R*_q_ = 0.52 nm, *R*_a_ = 0.38 nm) and ZnO after megasonic removal of ROP-patterned PVPh (*R*_q_ = 0.64 nm, *R*_a_ = 0.48 nm), which suggests that no significant amount of PVPh remains after the megasonic washing process. If any resist residues would remain after the washing process, then this does not appear to have any detrimental effect on the TFT electrical characteristics, as evidenced by good transfer and output characteristics and low contact resistance as presented and discussed in the latter sections.

In the ROP process, low-power oxygen (O_2_) plasma treatment (30 s, 0.25 mbar, 40 W, Diener Nano) was applied to enhance the wettability of the polymer ink on the PDMS blanket. The PVPh polymer ink was then applied on the PDMS blanket by using a capillary coater. When the polymer ink film reaches a semidry stage by partial solvent evaporation and partial absorption to PDMS, PDMS attached on the cylinder was rotated and it brought the polymer film into contact with a high-resolution relief printing plate with a positive raised image called a cliché. In the semidry stage, the polymer ink is both brittle enough to fracture under a nip pressure along the edges of the patterns on the relief plate and has good adhesion to the raised area of the cliché. This will lead to 100% ink transfer to the raised area (positive image) of the cliché. Then, the patterned semidry ink with the negative image remaining on PDMS is transferred onto a substrate. In both previous steps, no ink bleeding occurred in the semidry ink layer. This is the key strategy compared to other conventional printing methods, which resulted in sharp edges and vertical side walls that offer patterning of the layer at high resolution (down to single μm-level). Polymer resist printing was carried out with a sheet-fed type ROP machine (Jemflex Nihon Denshi Seiki Co., Ltd.) in controlled temperature and humidity conditions. The multilayer structure of TFTs was printed with a high overlay accuracy by using an automated alignment system.^47^ The ROP-patterned resist was utilized in LO and wet etching processes to pattern G and S/D electrodes and the SC layer, respectively. The ROP process is described in more detail in our previous article.^24^

### Electrode Patterning by Lift-Off

Negative images of G and S/D electrodes were formed on a substrate by printing a sacrificial polymer resist by ROP, as shown in Figure 2a,g. The printed resist was dried at room temperature for at least 10 min, resulting in a final dried film thickness of ∼60–70 nm. Then, the metal electrode material was vacuum-evaporated over the entire substrate, covering both the patterned resist and the exposed areas (Minilab ET080A, Moorfield Nanotechnology Ltd.). In this study, 6 nm titanium (Ti) and 24 nm gold (Au) were applied as bottom gate materials, where a thin Ti film acted as an adhesion layer. Correspondingly, either 40 nm aluminum (Al) or 20 nm Ti and 20 nm Au were used as S/D contact materials, where Ti acted as a low-work-function, nonalloyed Ohmic metal contact.^48^ Ti and Al were deposited by using e-beam evaporation, whereas Au was thermally evaporated. After evaporation, the LO process was then processed in a Megasonic bath (SONOSYS 1 × 8″, 1 MHz/50 Ohm/2000 W) containing methanol for 2 min to dissolve the resist, leaving the patterned G and S/D electrodes remaining on the substrate, as shown in Figure 2b,h.

**Figure 2:**
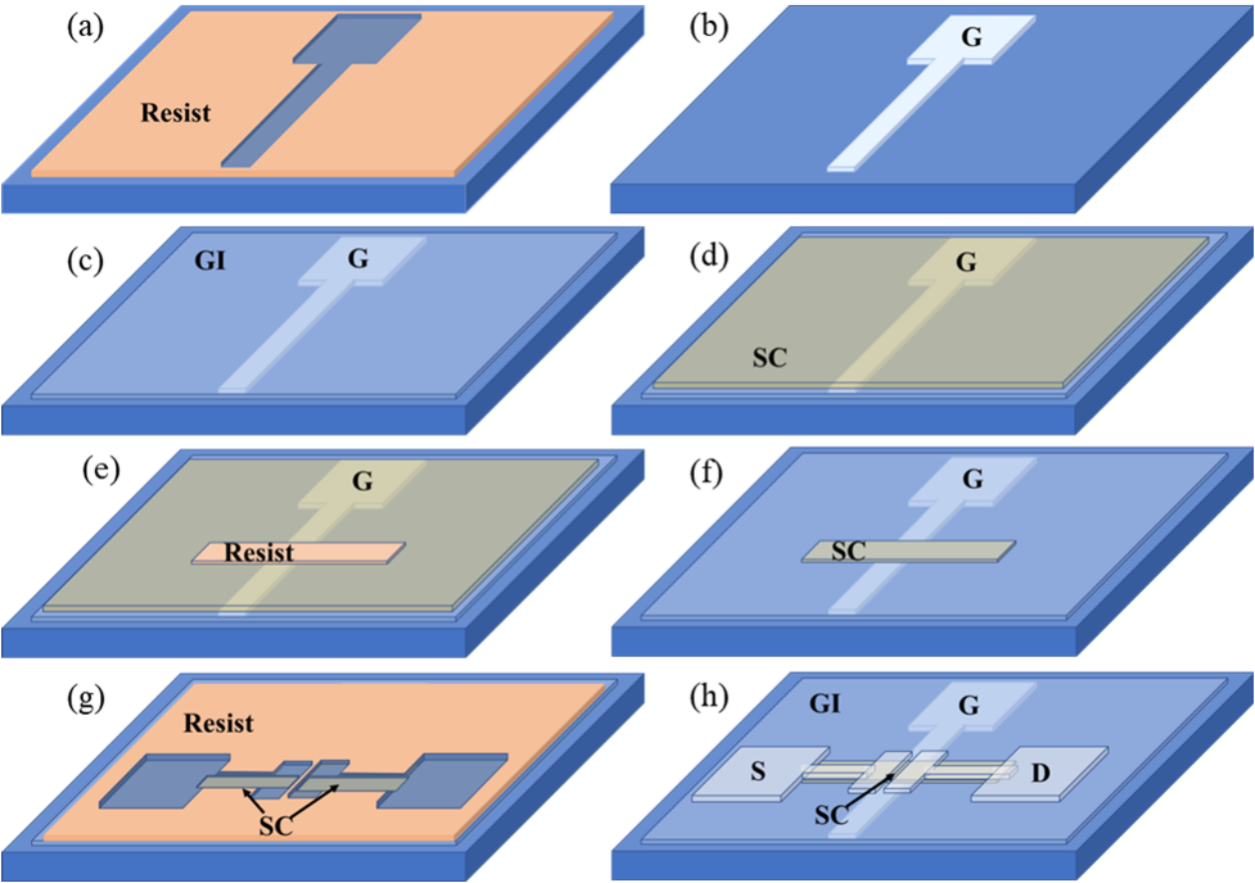
Schematic of the TFT fabrication process with ROP patterning. (a, b) G electrode patterning by resist printing and lift-off. (c, d) ALD-grown Al_2_O_3_ GI and ZnO SC layers. (e, f) SC patterning process by resist printing and etching. (g, h) S/D patterning by resist printing and lift-off.

### SC Patterning by Acidic Etching

In this work, GI and SC layers were grown on top of the patterned gate by ALD in the same run to preserve the interface between the GI and the SC.^49^ The layer arrangements of the GI and SC are shown in Figure 2c,d. ∼25 nm of Al_2_O_3_ (permittivity ε = 8.3) was first grown as the GI using trimethyl aluminum (TMA) and water (H_2_O) as the precursors followed by ∼9.1 nm ZnO deposited using diethyl zinc (DEZ) and H_2_O as precursors. The films were produced in a Beneq TFS500 thermal reactor in a single wafer reactor chalice at 150 °C. The carrier gas used was N_2_ supplied from an LN tank and dried with a typical purity of 99.9999%. Both DEZ and TMA are of electronic grade with 99.999+% metal purity. Precursors were kept at room temperature. The TMA and H_2_O process is composed of 0.2 s TMA pulse, 1 s purge, 0.2 s H_2_O pulse, and 2 s purge repeated for 250 cycles. The DEZ and H_2_O process is composed of 0.6 s DEZ pulse, 8 s purge, 0.25 s H_2_O pulse, and 5 s purge repeated for 51 cycles. The SC thickness of ∼9.1 nm was estimated from a separate calibration run composed of 6 cycles of the TMA and H_2_O process followed by 44 cycles of the DEZ and H_2_O process performed on a premeasured calibration monitor chip. The results of the optical model are presented in Figure S2. The polymer resist layer was printed with ROP on top of the freshly grown ZnO layer and dried at 150 °C for 10 min. At that drying temperature, the polymer resist tolerates mild acids and can be used to protect the ZnO layer below. The exposed ZnO area was etched away by 0.2 M oxalic acid (pH 1.5) for 15 s, while ZnO under the resist layer remained intact, as shown in Figure 2e. The protective resist layer on top of the patterned ZnO layer was then removed by dissolving in methanol using sonication (60 s), as presented in Figure 2f, and the sample was cleaned by a Megasonic bath in distilled water (DIW) for 30 s.

The selection of the etchant and optimization of etching parameters for the SC were conducted considering the requirements and characteristics of both the SC and the polymer resist. The polymer resist was tolerant of 0.2 M oxalic acid, and the exposed ZnO layer was totally etched away within 30 s. The final patterned ZnO layer after resist dissolution is shown in Figure S3c. However, some overetching of the SC can occur if the etching time is too long. First, the etching occurs at exposed areas. After a prolonged treatment time, the etching proceeds in the ZnO layer under the resist, resulting in the etchant penetrating inward under the resist. This overetching is proved by the narrowing of the SC lines as seen in Figure S4 when using 30 s etching time, where the overetching ranges from 0.8 to 1.05 μm per edge. Nevertheless, high-resolution line and space (*L*/*S*) patterning down to ∼4 μm can be achieved using the ROP-patterning technique, which is shown in Figure 3a–c. To limit the overetching, a shorter treatment time of 15 s was selected for the SC patterning in the TFT fabrication. Figure S5 shows the well-defined patterned SC on top of the G and GI layers after 15 s of etching before and after resist removal. Narrowing of the patterned SC lines was negligible after 15 s of etching.

**Figure 3:**
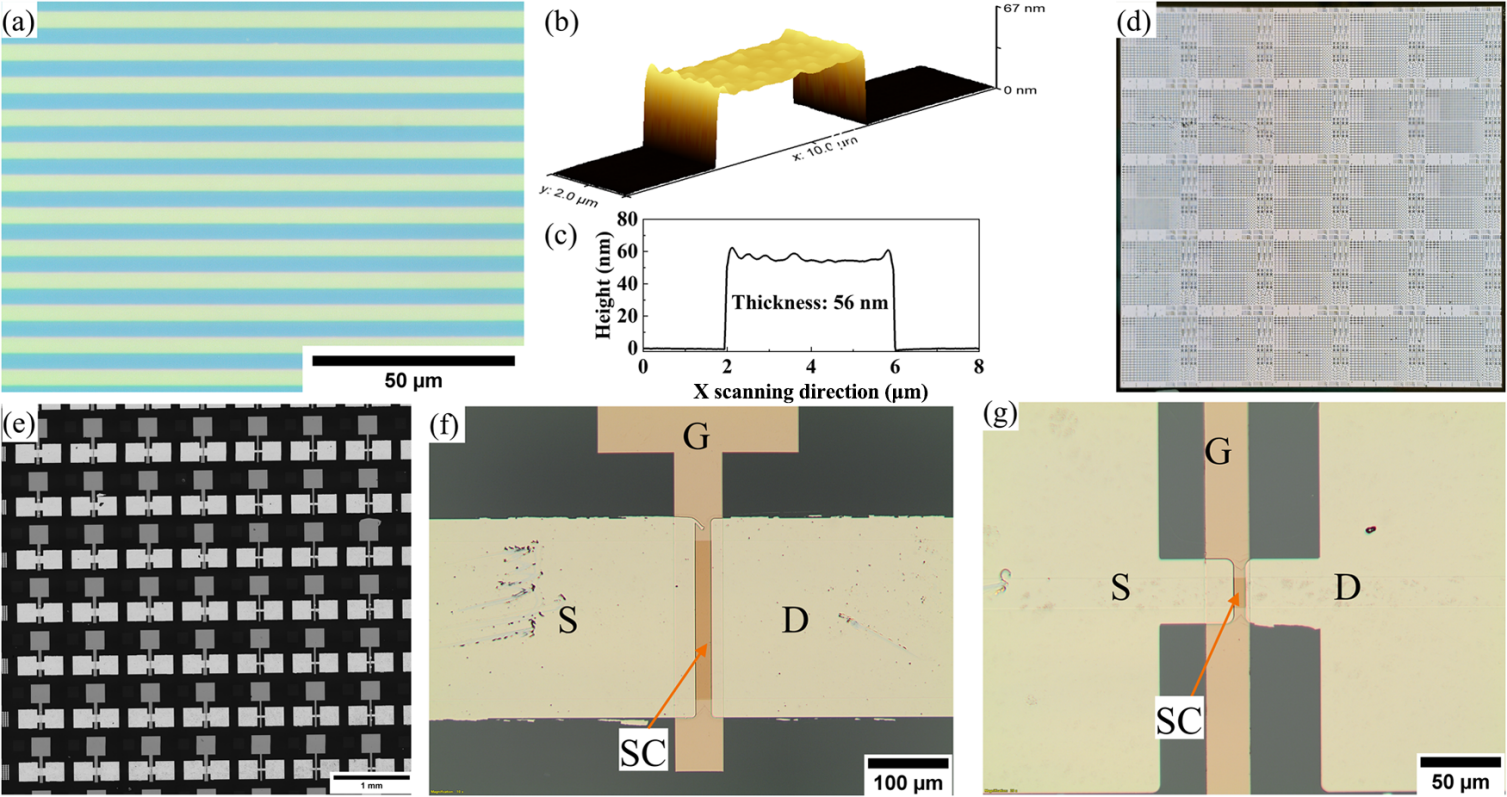
(a) Optical microscopy image of the printed resist 4 μm *L*/*S* structure and (b, c) AFM image and height profile over the 4 μm line. Optical microscopy images of ZnO TFTs with Ti/Au S/D: (d) 10 cm × 10 cm sample with 2 cm × 2 cm reticles, (e) TFT array, (f) *W* = 200 μm/*L* = 20 μm, and (g) *W* = 20 μm/*L* = 7.5 μm.

### ROP-Patterned ZnO TFTs

Figure 3d–g show the TFT structure that was ROP-patterned on a glass substrate with a size of 10 cm × 10 cm containing 25 dies each with a size of 2 cm × 2 cm. A high device density was realized, with each die containing 432 TFTs with different channel dimensions (nominal sizes ranging from *W* = 1 to 200 μm and *L* = 1 to 40 μm). The ROP patterning was successful in scaling the *L* of TFT down to the ∼5 μm level. The bottom G electrode and SC layer are well patterned with sharp edges. However, the patterning of S/D electrodes is not yet optimal. Both the leading and trailing edges of the patterns are slightly ragged, as seen in Figure 3f. This could possibly be attributed to the synchronizing issue of printing plate and PDMS cylinder or the ink being too dry during removal by the cliché. Normally, in ROP printing, machine direction (MD) alignment errors are greater than in the cross direction (CD) one due to the challenging control of the synchronization accuracy between a rotating cylinder and laterally moving stages under a nip pressure against a compressible PDMS printing blanket.^50,51^ In the MD seen as the vertical direction in Figure 3f, the alignment of the S/D electrodes is accurate, well below the typically observed MD alignment error for our current process that has achieved an accuracy of <3 μm in the MD and <2 μm in the CD over a 10 cm × 10 cm area. However, a slight misalignment is observed in the CD, seen as the horizontal direction in Figure 3g, but it is still within the tolerances specified in the layout design. This minor CD misalignment does not affect the measured electrical parameters that are measured at DC frequencies. The line structure of the SC shown in Figure 3f was employed to minimize the influence of MD misalignments of the printing machine. In this layout, the W is defined by the SC width, and the *L* is defined by the S/D gap; thus, no channel underlap exists that would lead to mobility overestimation.^52^ In the CD direction, a minor misalignment is allowed only if the S/D channel remains on top of the G electrode. No peeling of S/D electrodes was observed after probing, as shown in Figure 3f, which indicates a good adhesion between the Ti/Au S/D layer and the SC and GI layers.^48^

### Characterization

Grazing incidence X-ray diffraction (GIXRD) and X-ray reflectivity (XRR) were performed with a Rigaku SmartLab X-ray diffractometer. AFM images were taken with a Dimension 3100 SPM instrument from Digital Instruments. X-ray photoelectron spectroscopy (XPS) measurements were performed using a PHI Quantum3000 system equipped with an Al Kα X-ray source, a 46.95 eV pass energy, and a 0.2 eV step. Ar sputtering was done using 500 V for 24 s. Optical fittings of the calibration witness silicon chip were performed in a Semilab SE2000 ellipsometer system. Electrical characterizations of TFTs were performed with a Keithley 4200 SCS in the dark and in air at room temperature (temperature (*T*) ∼ 22–24 °C/relative humidity (RH) ∼ 38–50%). Negative bias illumination stress measurement was performed using a green LED light source (565 nm, M565L3, Thorlabs) with a 1.4 μW/cm^2^ light intensity.

## Results and Discussion

First, the ZnO thin film was grown with ALD on a silicon (Si) chip to study the properties of the ZnO thin films. GIXRD was implemented to characterize the microstructure of the ZnO film. The result shown in Figure 4a reveals clear evidence of polycrystallinity, with three peaks assigned to (100), (002), and (101) at around 31.8, 34.5, and 36.2°, respectively, which are in agreement with previous results.^53^ The measured and fitted XRR data of the same film are presented in Figure 4b. The inset shows the thin-film layer structure used in the fitting containing two ZnO thin films with different thicknesses and densities. This two-layer model gave a considerably better fit compared to a single ZnO layer. The thickness of the ALD-grown ZnO thin film on the Si chip obtained with XRR is ∼5.6 nm, which is close to the thickness obtained from the ellipsometer fitting (∼7.8 nm) within the same ALD run shown in Figure S2. The calculated average density (ρ_aveg_) of the ZnO film is ∼5.49 g/cm^3^, which is ∼98% of the ZnO powder density of 5.60 g/cm^3^.^54^ The density of the ALD-grown ZnO thin film is in agreement with the previously reported ALD-grown ZnO thin film at 150 °C,^42^ which is higher than the spin-coated ZnO thin film annealed at 200 °C containing a more amorphous structure^55^ and another solution-processed metal oxide SC thin film.^56^ Figure 4c shows the surface morphology of the patterned ZnO thin film on top of the Al_2_O_3_ film in the TFT actual devices with a thicker ZnO layer (see below), in which the thickness and average surface roughness (*R*_a_) are ∼13 and 0.425 nm, respectively. This indicates that a smooth surface, low edge raggedness, and well-defined vertical side walls are achieved using the ROP-patterning process. XPS measurements were performed to gain insight into the chemical environment of ALD-grown ZnO, both at the surface and after sputtering into the bulk ZnO film. The C 1s spectra, as displayed in Figure 4d, show a pronounced C–C signature peak at the surface of the ZnO film which disappears upon sputtering. This suggests the accumulation of adventitious carbon impurities on the sample surface, motivating the requirement for surface cleaning prior to any subsequent processing steps. Figure 4e,f presents the O 1s spectra before and after sputtering, deconvoluted into peak positions of components reported for similar materials.^42,57,58^ A higher binding energy component (∼531.5 eV surface/∼532.1 eV sputtered) is attributed to oxygen species that are not Zn–O lattice bonds, such as oxygen-deficient sites in the lattice, hydroxylation, and adsorbed H_2_O, CO_2_, or O_2_. The presence of adsorbate impurities is supported by a lower binding energy peak at around 530.6 eV assigned to Zn–O bonds of the oxide lattice, where the contribution of this component is smaller at the surface (∼37.9%) than in the bulk of the material (∼75.8%). After sputtering into the bulk of the ZnO film, elemental distributions obtained from O 1s and Zn 2p_3_ XPS spectra present stoichiometries of 47.6 and 56.4 atomic percent for O and Zn, respectively. The O:Zn ratio of 0.84 indicates a possibility for the presence of oxygen vacancies within the material, which may contribute to the nonlattice component shown in Figure 4f.

**Figure 4:**
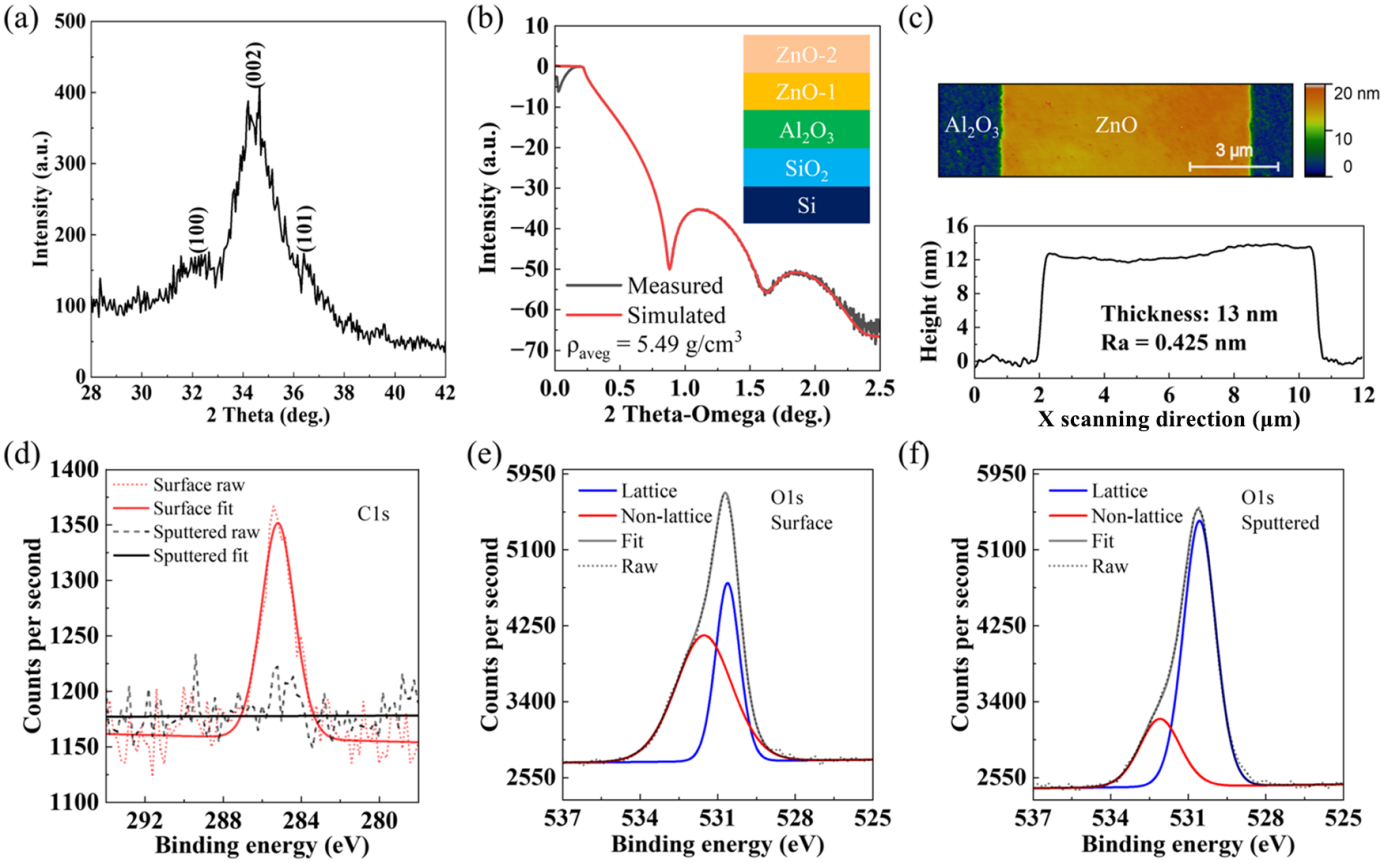
(a) GIXRD patterns and (b) XRR plots of ZnO thin films deposited by ALD on the Al_2_O_3_/Si substrate. (c) AFM image of the ROP-patterned ZnO thin film on top of the Al_2_O_3_/glass substrate in actual TFT devices. XPS (d) C 1s and (e) O 1s spectra of the ALD-grown ZnO surface and (f) after sputtering into the bulk film.

ZnO TFTs were then fabricated by using combined ALD and ROP-patterning processes on glass substrates. Figure 5a–d present the current/voltage (*I*/*V*) transfer characteristics of ALD-grown ROP-patterned ZnO-based TFTs measured after various durations of storage in the dark. All of these *I*/*V* curves exhibit much more stable *I*_on_/*I*_off_ and lower *I*_off_ of ∼10^–13^ A when compared to the nonpatterned semiconductor TFTs with *I*_off_ of ∼10^–9^ A.^24^ The detailed and calculated electrical parameters, including μ_FE_, *V*_on_, threshold voltage (*V*_t_), *V*_hyst_, *I*_on_/*I*_off_, and subthreshold slope (SS), based on their *I*/*V* curves are summarized in Table 1. μ_FE_ in the linear region can be calculated based on the ideal square-law model shown in eq 1:^52^

1 $$\mu_{\mathrm{FE}}=\frac{\frac{\partial I_{d}}{\partial V_{g}}}{\frac{W}{L}C_{\mathrm{ox}}V_{d}}$$

where *I*_d_ is the drain current, *V*_g_ is the gate voltage, *V*_d_ is the drain voltage, and *C*_ox_ is the gate capacitance per unit area. Compared to μ_FE_ and *V*_on_ measured on the day of device fabrication, an obvious improvement was observed for those measured after 59 days, indicating stabilization of the device performance during aging. In addition, it is found that the TFTs exhibit a neglectable clockwise *V*_hyst_ ∼0.13 V and a small SS ∼140 mV/dec, which implies only a small amount of charge traps existing at the interface between GI and SC layers.^53^ Moreover, Figure 5e presents the output curve of a typical ZnO TFT with Ti/Au S/D (*W* = 200 μm/*L* = 20 μm), which shows good saturation of *I*_d_. The corresponding calculated μ_FE_ of a typical TFT shown in Figure 5f is ∼16.6 cm^2^ (Vs)^−1^. Additionally, the output curves of a typical ZnO TFT selected from Figure 5 measured after different days at a low-*V*_d_ range are shown in Figure S6, in which no upward curvature is observed, indicating no sign of a Schottky-type contact.^19,59^ This linear *I*/*V* relation suggests an Ohmic-like contact behavior and good charge injection in S/D electrodes.^60^ Al was also investigated as the S/D electrode in this work. Figure S7 shows the *I*/*V* transfer characteristic of Al S/D-based ZnO TFTs. As listed in Tables 1 and S3, it is worth noting that all of the electrical parameters from devices with Ti/Au S/D are superior to those with Al S/D. Additionally, a Schottky-type output curve of ZnO TFTs with Al S/D was observed in Figure S7b,c. Therefore, we selected Ti/Au as S/D electrodes for further characterizations in the following sections.

**Figure 5:**
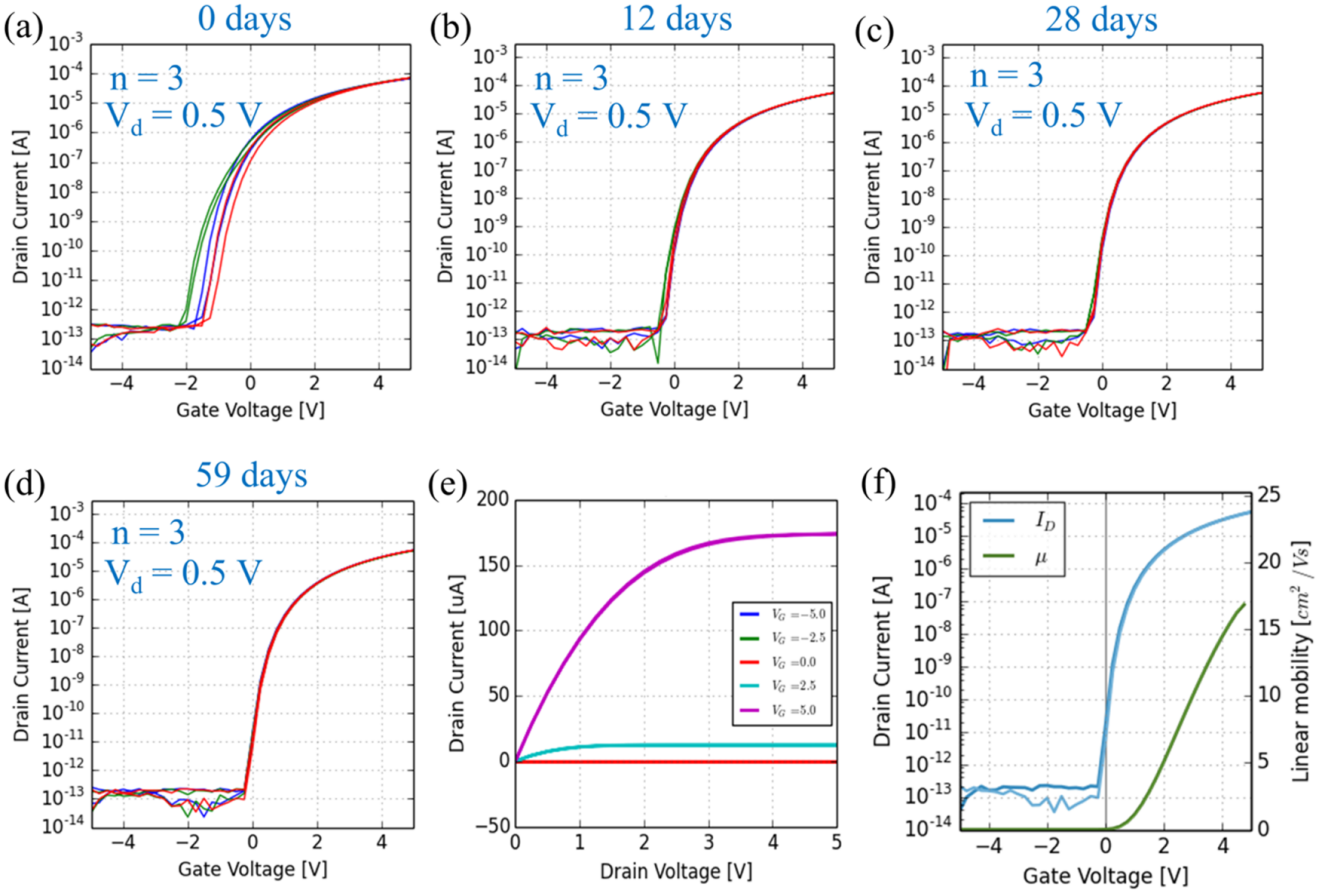
Transfer curves of ALD-grown ROP-patterned ZnO TFTs with Ti/Au S/D (*W* = 200 μm/*L* = 20 μm) measured after (a) 0, (b) 12, (c) 28, and (d) 59 days. (e) Output curve and (f) transfer curve with the calculated linear mobility of a typical ZnO TFT based on panel (d).

**Table 1:** Detailed Electrical Characterization Parameters Calculated Based on Data from Figures 5a–d and 8a^a^

ZnO TFTs with titanium/gold (Ti/Au) S/D					
	0 days	12 days	28 days	59 days	27 days
number of working TFTs (*n*)	*n* = 3 (*n*_total_ = 3)				*n* = 8 (*n*_total_ = 12)
*W*/*L* (μm)	200/20				20/7.5
μ_FE_ (cm^2^(Vs)^−1^)	15 ± 2	16.4 ± 0.3	16.9 ± 0.3	16.6 ± 0.4	15.1 ± 0.8
*V*_on_ (V)	–2.0 ± 0.4	–0.7 ± 0.3	–0.75 ± 0.13	–0.49 ± 0.02	–0.49 ± 0.14
*V_t_* (V)	–0.9 ± 0.3	0.60 ± 0.05	0.52 ± 0.01	0.71 ± 0.02	0.6 ± 0.2
*V*_hyst_ (V)	0.24 ± 0.05	0.13 ± 0.05	0.13 ± 0.07	0.13 ± 0.04	0.13 ± 0.03
*I*_on_/*I*_off_	6 × 10^8^ ± 5 × 10^8^	3 × 10^8^ ± 3 × 10^7^	3 × 10^8^ ± 8 × 10^6^	2.8 × 10^8^ ± 1.1 × 10^7^	1 × 10^9^ ± 6 × 10^8^
SS (V/decade)	0.167 ± 0.013	0.13 ± 0.03	0.11 ± 0.03	0.14	0.11 ± 0.03

a *n*_total_: number of total fabricated TFTs.

TFTs with a constant channel width (*W* = 200 μm) and a varied channel length of *L* = 5, 10, 20, or 40 μm were explored to study the effects of scaling the channel length upon electrical performance metrics. Table S4 summarizes the detailed electrical parameters of these TFTs measured after 59 days. μ_FE_ of TFTs summarized in Table S4 was calculated based on eq 1. However, when *L* becomes shorter, the effect of contact resistance (*R*_S/D_) between the S/D electrodes and the SC will be evident. The *R*_S/D_-affected mobility in the linear region, called extrinsic charge mobility (μ_ext_), can be calculated based on eq 2.^52^

2 $$\mu_{\mathrm{ext}}=\frac{\mu_{\mathrm{in}}}{1+R_{S/D}\frac{W}{L}\mu_{\mathrm{in}}C_{\mathrm{ox}}\left(V_{g}-V_{t}\right)}$$

where *R*_S/D_ is the contact resistance from the source and drain regions, μ_in_ is the intrinsic field-effect mobility (that is independent of *R*_S/D_), and *V*_t_ is the threshold voltage determined from the linear extrapolation of the *I*_d_^1/2^/*V*_g_ transfer characteristic.^61^ μ_in_ can be calculated using the transfer length method (TLM), in which two equations need to be applied to obtain it:^62^

3 $$R_{\mathrm{total}}=r_{\mathrm{ch}}L+R_{S/D}$$

4 $$r_{\mathrm{ch}}=\frac{1}{\mu_{\mathrm{in}}C_{\mathrm{ox}}W\left(V_{g}-V_{\mathrm{ti}}\right)}$$

where *R*_total_ is the total resistance, *r*_ch_ is the channel resistivity, and *V*_ti_ is the intrinsic threshold voltage. Figure 6a exhibits the plots of ZnO TFTs at different *V*_g_ values with linear fits to the data. The fitting quality factors (*R* ^2^) shown in Table S5 are all ≥0.999, which indicates that the fitting is good. Nearly all of the fitted lines meet at one point in the first quadrant, which could suggest that a doped interface region between the S/D and the SC has been formed. This can be attributed to the oxygen-scavenging phenomenon because of a higher Gibbs free energy of Ti oxidation than for the SC.^48^ After the deposited metal has scavenged oxygen from ZnO, oxygen vacancies will be left inside the SC, which can act as charge donors, thus increasing the charge carrier concentration.^19^ Based on eq 3, the *R*_S/D_ and *r*_ch_ at different *V*_g_ values can be extracted from the interception of the *y*-axis and slope, respectively. The results of the line fits are summarized in Table S5. In principle, *R*_S/D_ should be positive; however, there are some negative *R*_S/D_ values obtained for the smaller *V*_g_ values. These can be attributed to the high *r*_ch_ ≥ 1190 Ω/μm of our SC between S/D at small gate fields (*V*_g_ ≤ 3.5 V), whose variation can introduce a high error in the *R*_S/D_ values.^63^ The thin SC with a high *r*_ch_ can also magnify this error when compared to the bulk (i.e., thick) SC.^63^ By plotting the 1/*r*_ch_ as a function of *V*_g_, as shown in Figure S8, based on eq 4, we can read μ_in_ ∼ 17.48 cm^2^ (Vs)^−1^ and *V*_ti_ ∼ 2.75 V from the slope and the *x*-intercept of the tangent line, respectively. For the μ_FE_ shown as a function of *L* in Figure 6b, the values are obtained at the largest ∂*I*_d_/∂*V*_g_ that occurs at *V*_g_ = 5 V (see Figure 5f). Therefore, when calculating the μ_ext_, *R*_S/D_ is obtained from the *y*-intercept of the line at *V*_g_ = 5 V, as shown in Figure 6a, which equals ∼260 Ω. The contact resistance normalized with the contact width (*R*_S/D_W), where *W* = 200 μm, is ∼5 Ω·cm. Ti/Au contacts that are patterned with ROP LO show lower *R*_S/D_W than for print-patterned contacts (∼160 Ω·cm for inkjet-printed Al-doped CdO S/D to the In_2_O_3_ semiconductor^19,64^ ) and are on par with *R*_S/D_W for vacuum-deposited contacts patterned with PL (from ∼1 Ω·cm upward to the IGZO semiconductor).^19^ This reasserts that PVPh resist residues are unlikely to be present at the ZnO–S/D interface after the presented process. Figure 6b shows both the calculated μ_FE_ and the μ_ext_ obtained from eq 2 as a function of *L*. It is obvious that μ_FE_ follows the trend of the calculated μ_ext_ plot. Regardless of the low *R*_S/D_W values obtained here, this indicates that the *R*_S/D_ does indeed decrease the device mobility in short *L* devices, which suggests that contact resistance should be considered when discussing the mobility of TFTs at the high-mobility/short-channel limit.

**Figure 6:**
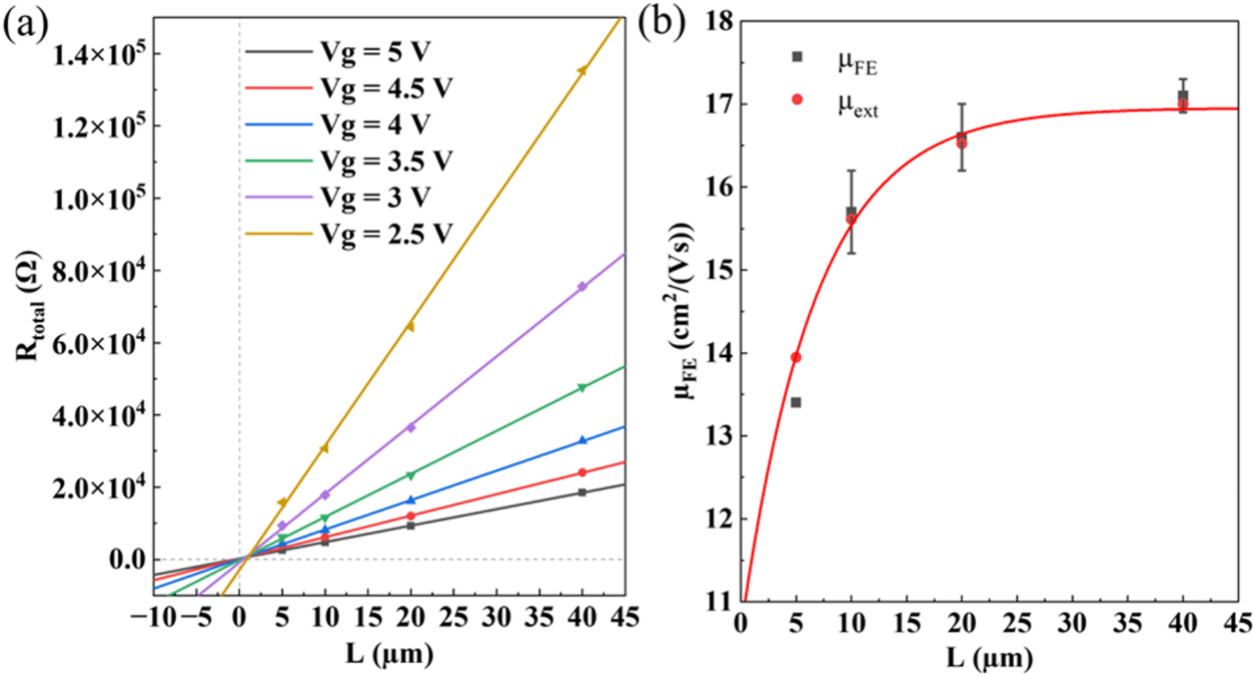
(a) TLM plots of ZnO TFTs with *R*_total_ as a function of different *L* at different *V*_g_ values. (b) Plot of μ_FE_ calculated from eq 1 and calculated μ_ext_ based on eq 2.

The operation stability of unpassivated ZnO TFTs was studied using positive bias stress (PBS), negative bias stress (NBS), and negative bias illumination stress (NBIS) measurements. It is known that complex phenomena such as charge trapping, defect generation, and interactions of ambient gases with the exposed back channel of unpassivated metal oxide TFTs can lead to shifts in the *V*_on_ during constant gate field bias conditions. Figure 7a–c show the corresponding *I*/*V* curves of the different bias conditions at the 2.2 MV/cm gate field (*E*). In NBIS, the light source was an LED with green light at a wavelength of 565 nm and a light intensity of 1.4 μW/cm^2^. Obvious positive and negative shifts of *V*_on_ can be observed in Figure 7a,c, respectively. The calculated shift of *V*_on_ as a function of the bias stress time for these bias stress conditions is shown in Figure 7d. The TFT under NBS shown in Figure 7b exhibits a neglectable shift of *V*_on_, indicating that the number of pre-existing hole traps is negligible and the desorption of O_2_ or adsorption of H_2_O under the negative gate field does not occur significantly.^65^ The positive shift of *V*_on_ shown in Figure 7a can be attributed to charge trapping, defect generation (as SS slightly deteriorates), or interactions with ambient gas molecules (adsorption of O_2_ or desorption of H_2_O) under PBS.^66,67^ The negative shift of *V*_on_ under illumination, as shown in Figure 7c, can possibly result from the light-promoted desorption of O_2_ from the back channel or the photoionization of oxygen vacancies (V_o_ → V_o_^2+^) and their migration to the GI/SC interface.^46^ Regardless of the operation mechanism, we expect that the stability of the devices could be improved by the deposition of a suitable encapsulation layer to prevent interactions of the back channel with ambient gas molecules.

**Figure 7:**
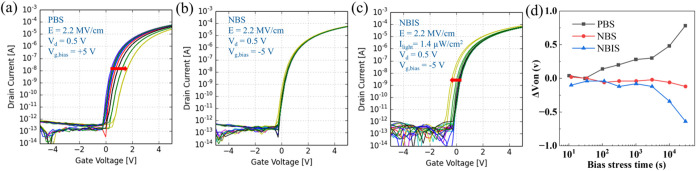
(a) PBS, (b) NBS, and (c) NBIS measurements of ALD-grown ROP-patterned ZnO TFTs with Ti/Au S/D (*W* = 200 μm/*L* = 20 μm). (d) Shift of *V*_on_ calculated from panels (a–c) as a function of the bias stress time.

Finally, we examined the smallest ALD-grown ZnO TFT device size that we could fabricate with our current ROP-patterning process. By considering the limits of overlay alignment accuracy and patterning resolution, as well as the effect of *R*_S/D_ in the electrical performance of the TFTs, the smallest devices that were successfully measured were *W* = 20 μm and *L* = 7.5 μm, as shown in Figure 3g. Figure 8a presents the *I*/*V* transfer characteristic of ALD-grown ROP-patterned ZnO with Ti/Au S/D and this small *W*/*L* ratio. Electrical parameters including μ_FE_, *V*_on_, *V*_t_, *V*_hyst_, *I*_on_/*I*_off_, and SS calculated based on Figure 8a are summarized in Table 1. The devices exhibit good uniformity, a relatively high μ_FE_ ∼15.1 cm^2^ (Vs)^−1^ (as shown in Figure 8c for a typical device), a high *I*_on_/*I*_off_ ratio >10^9^, a near zero *V*_on_ ∼−0.49 V, a negligible *V*_hyst_ ∼0.13 V, and a small SS ∼110 mV/dec. In addition, Figures 8b and S9 also demonstrate that the small-scale TFTs operate with Ohmic-like electrodes and exhibit a good saturation of *I*_d_. The recent advances in precision engineering demonstrate that 3σ = 1 μm position accuracy can be achieved on a wafer-scale sheet-based ROP process^68^ and also ∼1 μm level in a continuous R2R printing process (microcontact printing).^69^ These results suggest that if *R*_S/D_ can be minimized, for example, with interface engineering to not limit the μ_FE_ for the high-mobility and short-channel devices, ROP-patterned ALD-grown TFTs could be miniaturized below *L* < 5 μm using a scalable process.

**Figure 8:**
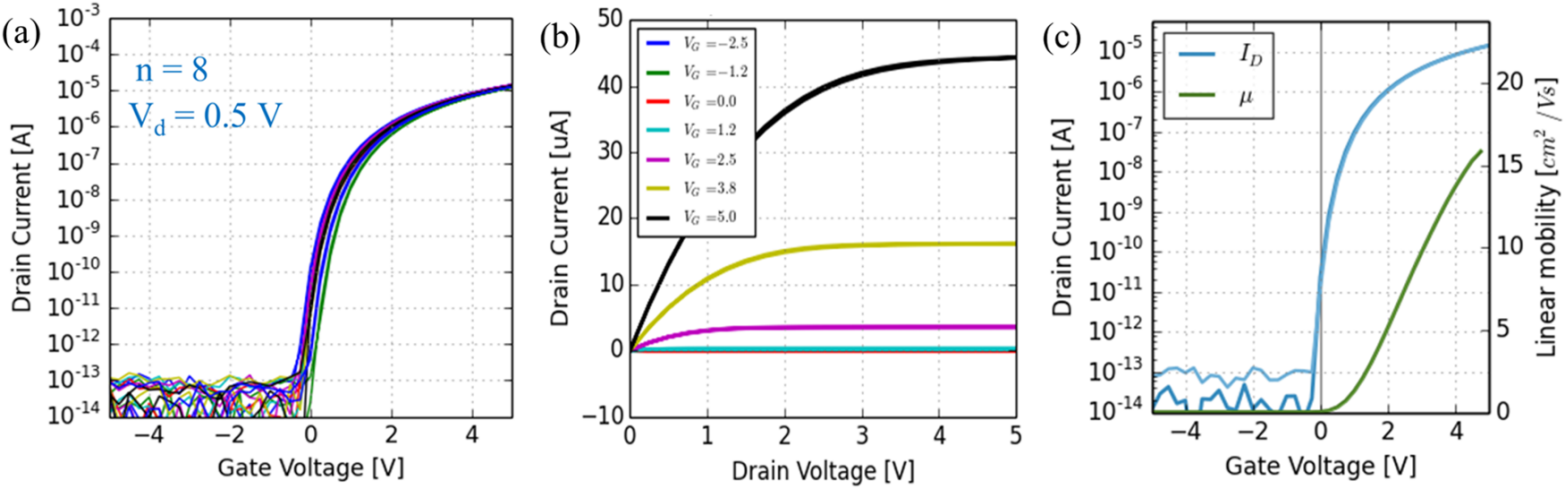
(a) Transfer curves of ALD-grown ROP-patterned ZnO TFTs with Ti/Au S/D (*W* = 20 μm/*L* = 7.5 μm). (b) Output curve and (c) transfer curve with the calculated linear mobility of a typical ZnO TFT from panel (a).

## Conclusions

ALD-grown Al_2_O_3_ and ZnO GI/SC stack and high-resolution ROP patterning are successfully combined and applied in the fabrication of high-performance ZnO TFTs with a low thermal budget of 150 °C. The ROP process enabled the patterning of the ZnO SC and high-quality vacuum-deposited Ohmic-like Ti/Au contact electrodes at μm-level resolution down to a 5 μm channel length. The fabricated TFTs exhibit promising uniformity and stability, a high μ_FE_ ∼16.6 cm^2^ (Vs)^−1^, an almost zero *V*_on_ ∼−0.49 V, a high *I*_on_/*I*_off_ ratio >10^8^, a low *V*_op_ of ≤5 V, a negligible *V*_hyst_ ∼0.13 V, and a small SS ∼140 mV/dec. In addition, miniaturized devices also exhibit a similar good electrical performance. In addition, we find that the decrease in the channel length of the devices leads to contact-resistance-limited operation when the contact resistance is at the same order of magnitude as the channel resistance. Thus, regardless of the Ohmic contact performance and low width-normalized contact resistance (∼5 Ω·cm) of the ROP-patterned Ti/Au contacts, attention to contact resistance must be given to improve the miniaturization of these TFTs. In the future, we expect that the ROP quality can be improved to increase the yield of small-scale (*W* and *L* ≤ 5 μm) devices. Additionally, the single multifunctional printed resist by ROP can be further investigated to pattern all TFT layers on a flexible substrate to enable the fabrication of TFT-based circuits. We believe that the combination of low-temperature ALD and ROP patterning can be further developed to provide an economical route for the fabrication of future metal oxide semiconductor-based electronics to be applied in various applications, ranging from flexible electronics to wearable electronics and biodegradable sensor systems.

## Supplementary Material



## Notes

Additional tables related to state of art in ZnO TFTs and different fabrication methods for TFTs, additional figures related to spectroscopic ellipsometry of ALD fabrication, ROP patterning, surface characterization after resist removal, electrical characterization and fitting, tables for calculated and fitted electrical parameters related to Al S/D ZnO TFTs and Ti/Au ZnO TFTs with different *W*/*L* scales, and results of the line fits of TLM (DOCX)
