# Supplementary material for: ALD-Grown ZnO TFTs Patterned by High-Resolution Reverse-Offset Printing
Source: ACS Appl Mater Interfaces. 2025 Jun 3;17(23):34150–60. doi: 10.1021/acsami.5c03321 (PMC12163932; doi:10.1021/acsami.5c03321)
Supplement: Supplementary file 1 [file am5c03321_si_002.docx]

**Supporting information:**

ALD-grown ZnO TFTs patterned by high-resolution reverse-offset printing

Fei Liu ‡*, Asko Sneck ‡, Patrik Eskelinen, Olli Halonen, Liam Gillan, Jaakko Leppäniemi

VTT Technical Research Centre of Finland Ltd, Espoo, Uusimaa, 02150, Finland

* [fei.liu@vtt.fi](mailto:fei.liu@vtt.fi)

**Table S1.** Reported ZnO TFTs with different deposition (dep.) and patterning (patt.) methods. T = process temperature; W/L = channel width/length; S/D = source/drain electrode (material); µ = electron mobility; V_g,range_ = range of applied gate voltage; V_t_ = threshold voltage; I_on_/I_off_ = current ON/OFF-ratio; V_hyst_ = voltage hysteresis in transfer curve; PL = photolithography; SPU = sputtering; Ev = evaporation; SM = shadow mask; RT = room temperature; EHD = electrohydrodynamic jet (printing); LO = lift-off; ALD = atomic layer deposition.

| **ZnO semiconductor** | | | **Source/drain electrodes** | | | **Device performance** | | | | | **Ref.** |
| --- | --- | --- | --- | --- | --- | --- | --- | --- | --- | --- | --- |
| **Deposition** | **T (°C)** | **Patt.** | **W/L (µm)** | **S/D** | **Dep. /patt.** | **µ**  **(cm^2^ (Vs)^-1^)** | **V_g, range_ (V)** | **V_t_**  **(V)** | **I_on_/ I_off_** | **V_hyst_ (V)** |  |
| **Solution process (incl. printing)** | | | | | | | | | | | |
| Spray pyrolysis | 350 | PL | 20/3 | IZO | SPU/- | 5.35 ± 3.80 | -5 to 5 | 0.83± 0.06 | ~10^5^ | - | ^1^ |
| Spray pyrolysis | 350 | PL + wet-etch | 50/10 | Mo | SPU/ PL + dry-etch | 11.48 ± 2.37 | -5 to 7 | 2.8 ± 0.15 | ~10^8^ | - | ^2^ |
| Spray pyrolysis | 400 | - | 2000/ 20 | Al | Ev/SM | ~10 | -2 to 10 | - | ~10^6^ | - | ^3^ |
| Spin coating | 300 | - | 1000/ 100 | Al | Ev/SM | 1.28 | -30 to 30 | 7.63 | ~10^8^ | - | ^4^ |
| Spin coating | 300 | - | 2000/ 100 | Al | Ev/SM | ~11 | -40 to 80 | - | >10^7^ | - | ^5^ |
| Spin coating | 210 | - | - | Al | Ev/SM | 1.37 ± 0.09 | -20 to 80 | 8.81 ± 1.51 | ~10^6^ | - | ^6^ |
| Spin coating | 400 | - | 1000/ 100 | Al | Ev/SM | 2.6 | -20 to 100 | 23.1 | ~10^2^ | - | ^7^ |
| Spin coating | 300 | - | 800/ 50 | Al | Ev/SM | 1.85 | -100 to 100 | 25.8 | ~10^7a)^ | - | ^8^ |
| Printing (Aerosol-jet) | 300 | - | 200/ 50 | Au/ Cr | -/PL | 1.9 ± 0.2 | -1 to 1 | 0.5 ± 0.1 | ~10^5^ | ~0.2^a)^ | ^9^ |
| Printing (Inkjet) | 300 | - | ~200/70 | Al | Ev/SM | 0.263 | -20 to 20 | - | ~10^5^ | - | ^10^ |
| Printing (Screen) | RT | - | 500/ 200 | C paste | Screen print/ mold | 21.7 ± 2.70 | -0.5 to 2.5 | - | ~10^4^ | ~0.5^a)^ | ^11^ |
| Printing (EHD) | 450 | - | 15000/600 | Al | Ev/SM | 0.06 ± 0.01 | -20 to 40 | 17.57 ± 5.45 | ~10^4^ | - | ^12^ |
| **Vacuum deposition** | | | | | | | | | | | |
| SPU | 80 | - | - | Al | SPU/ PL + wet-etch | 0.27 | -20 to 50 | 25.45 | ~10^3^ | - | ^13^ |
| SPU | 300 | PL + LO | 100/ 10 | ITO | SPU/PL + LO | 0.15 | -10 to 20 | - | ~10^5^ | - | ^14^ |
| SPU | 200 | - | 1000/30 | Al | Ev/SM | ~1.6 | -10 to 20 | ~5 | ~10^7^ | - | ^15^ |
| SPU | - | - | 800/ 50 | Mo | SPU/- | ~1.3 | -1 to 9^a)^ | ~2.3 | ~10^6^ | - | ^16^ |
| ALD | 150 | - | 1000/80 | Cr/ Au | SPU/- | 12.4 | -5 to 10 | 0.28 | ~10^7^ | - | ^17^ |
| ALD | 200 | PL + oxide etch | 10/5 | Ti/Pt | Ev/PL + LO | 85 | -2 to 5 | 0.72 | ~10^8^ | <0.05 | ^18^ |
| ALD | 200 | PL + wet-etch | 5/5 | ITO | SPU/PL + LO | 43.8 ± 0.80 | -10 to 15 | 2.73 ± 0.02 | >10^9^ | <0.01 | ^19^ |
| ALD | 200 | PL + LO | 40/10 | Ni | Ev/PL + LO | ~32.8 | -2 to 7 | ~1.2^a)^ | ~10^12^ | - | ^20^ |
| ALD | 100 | PL + wet-etch | 50/3 | Cu/Ti | Ev/PL + LO | 45.3 | -5 to 25 | 1.126 | ~10^9^ | - | ^21^ |
| ALD | 100 | PL + LO | 200/ 50 | Au/ Cr | Ev/PL + LO | 32.1 | -10 to 20 | 1 ± 0.05 | 10^8^ | - | ^22^ |
| ALD | 400 | - | 5/5 | ITO | SPU/ PL + LO | 17.9 | -10 to 15 | 3.23 | ~10^10^ | 0.02 | ^23^ |
| **Vacuum deposition with print-patterning** | | | | | | | | | | | |
| ALD | 200 | Inkjet | 400/ 100 | AZO | ALD/ Inkjet | 3 | -5 to 15 | - | ~10^4a)^ | - | ^24^ |
| ALD | 150 | ROP + etch | 20/7.5 | Ti/Au | Ev/ROP + LO | 15.1 ± 0.8 | -5 to 5 | 0.6 ± 0.2 | >10^8^ | 0.13 ± 0.03 | Our work |

1. Value estimated from figure inside reference paper.

**Table S2.** Comparison of estimated performance of different deposition and patterning methods in TFT fabrication process. G = gate; GI = gate insulator; SC = semiconductor; SD = source/drain; LER = line edge roughness; EHD = electrohydrodynamic jet printing; SIJ = super inkjet printing; UPD = ultra-precise dispensing; ROP = reverse offset printing; µCP = Microcontact printing; PL = photolithography.

| **Deposition/ patterning method** | **Patterning during deposition** | **High-resolution & low LER** | **Scalability to large area/ volume** | **Optimal for TFT layer** | **Uniform layer thickness** | **Multi-layer (incl. align-ment)** | **Wide material palette** |
| --- | --- | --- | --- | --- | --- | --- | --- |
| **Printing processes** | | | | | | | |
| Inkjet printing | Yes |  | High | G/GI/SC/SD |  |  |  |
| Flexography | Yes |  | High | GI/SC |  |  |  |
| Gravure printing | Yes |  | High | GI/SC |  |  |  |
| Screen printing | Yes |  | High | - |  |  |  |
| EHD/SIJ/UPD | Yes | Yes | Poor | G/SD |  |  |  |
| ROP | Yes | Yes | High | G/SD | Yes | Good |  |
| **Vacuum deposition and patterning** | | | | | | | |
| µCP + deposition |  | Yes | High | G/SD | Yes | Good |  |
| ROP + deposition |  | Yes | Moderate | G/GI/SC/SD | Yes | Good | Yes |
| PL + deposition |  | Yes | Poor | G/GI/SC/SD | Yes | Good | Yes |

**
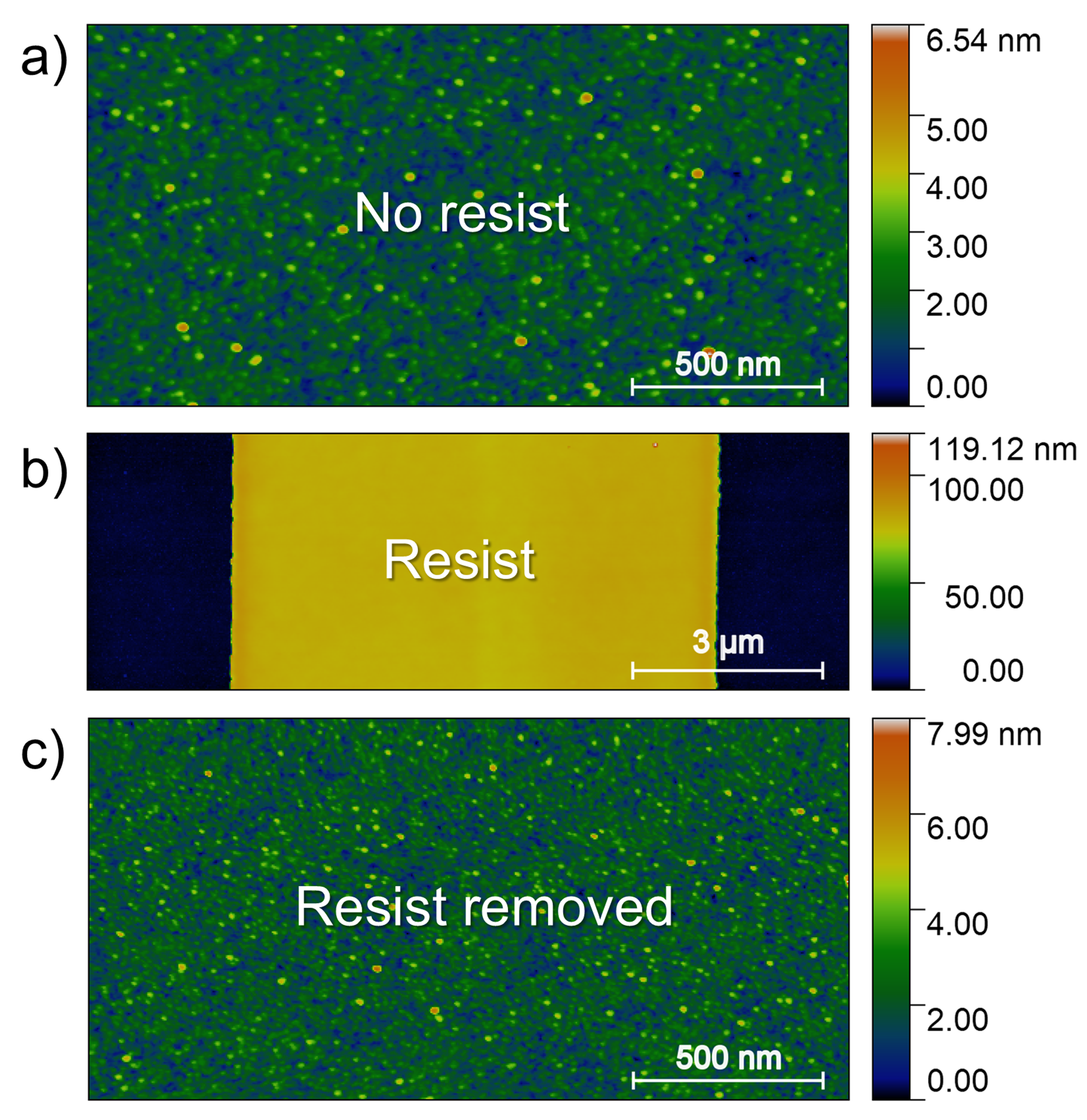
**

**Figure S1.** AFM data revealing similar surface root mean square roughness (R_q_) and average roughness (R_a_) for a) ZnO with no PVPh (R_q_ = 0.52 nm, R_a_ = 0.38 nm), b) ZnO with ROP patterned PVPh (measurements taken from ZnO regions devoid of PVPh, R_q_ = 0.53 nm, R_a_ = 0.39 nm), and c) ZnO after megasonic removal of PVPh (R_q_ = 0.64 nm, R_a_ = 0.48 nm).

**
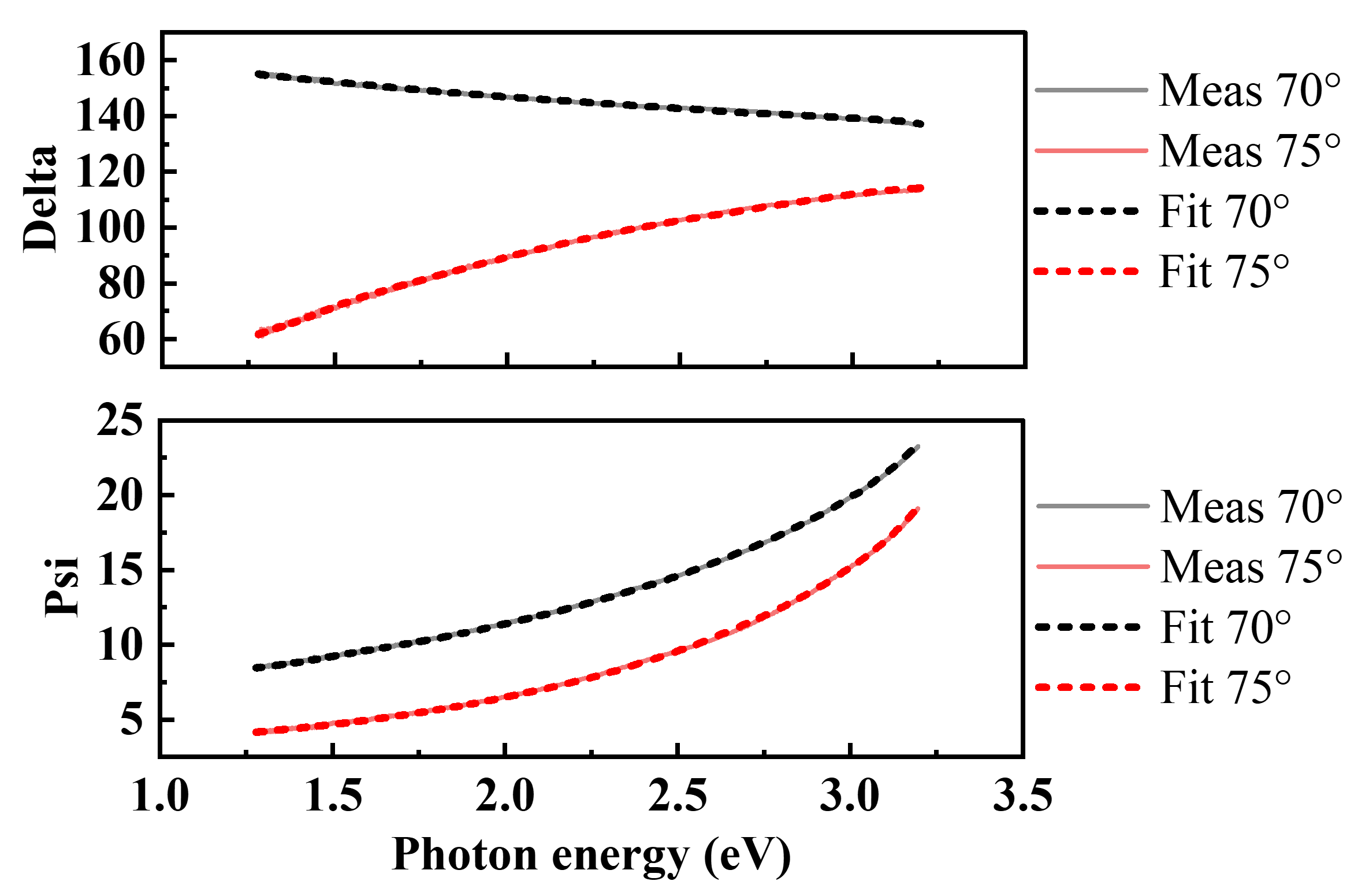
**

**Figure S2.** Spectroscopic ellipsometer Psi and Delta measurements and optical fittings of calibration witness silicon chip.

Witness chip measured prior to deposition to determine native oxide. For optical model native oxide is fixed at 1.164 nm as determined prior. A fixed 0.6 nm Al_2_O_3_ film is also used. The measurement is performed at both 70- and 75-degree incidence angle in a Semilab SE2000 ellipsometer system. The ZnO layer is modelled using a Cauchy dispersion model fitting for A and B and thickness in the photon energy range of 1.28 to 3.2 eV. The respective values for the fits are A – 1.83909, B – 0.018101 and thickness 7.811 nm. A relevant description of the approach used is available in ^25^.


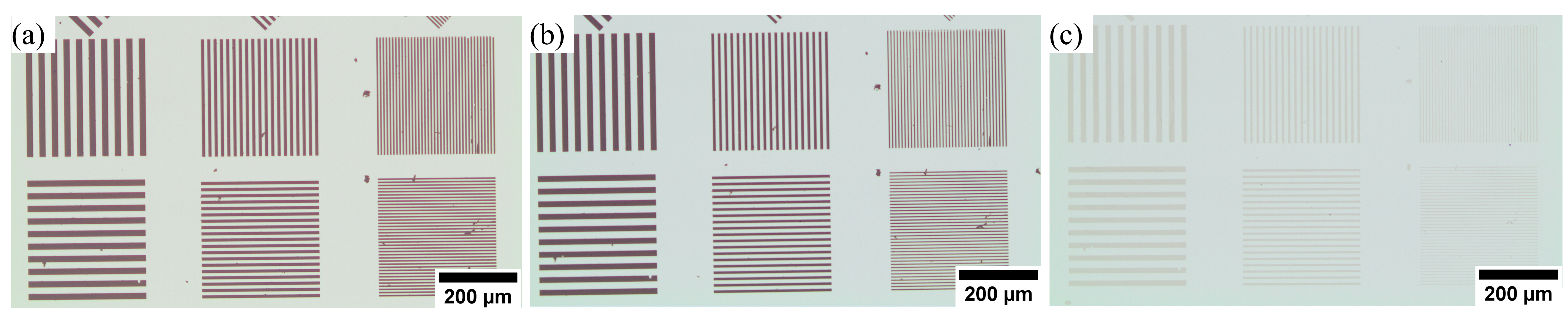


**Figure S3.** Optical microscopy images of 16, 8 and 4 µm line/space (L/S) patterns: (a) after resist printing, (b) after 30 s oxalic acid etching and (c) after dissolving the resist showing the final patterned ZnO structure.


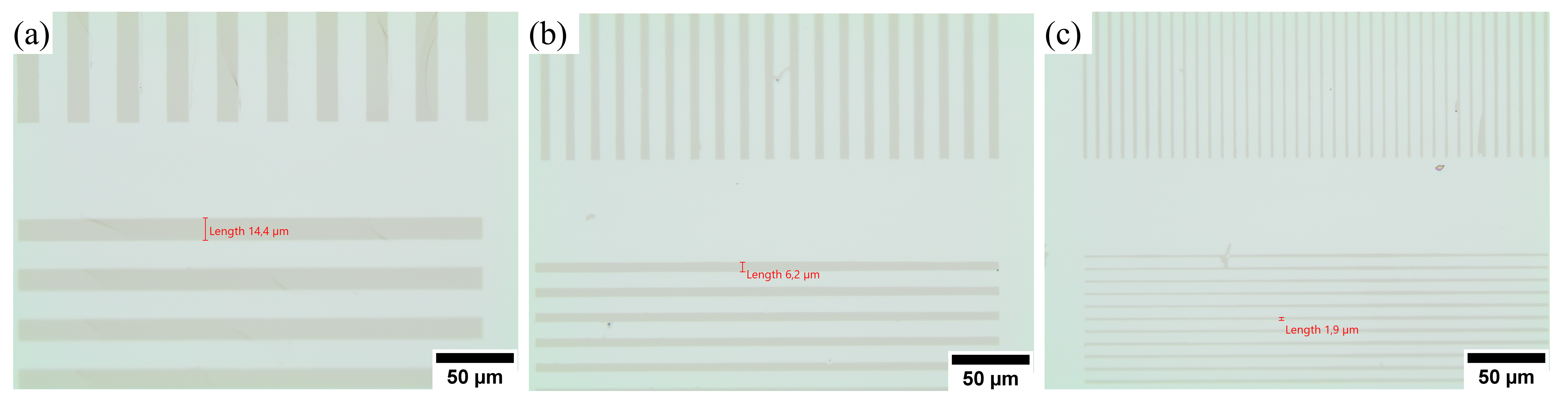


**Figure S4.** Optical microcopy images of L/S patterns with nominal widths of (a) 16, (b) 8 and (c) 4 µm after 30 s oxalic acid etching and resist removal.


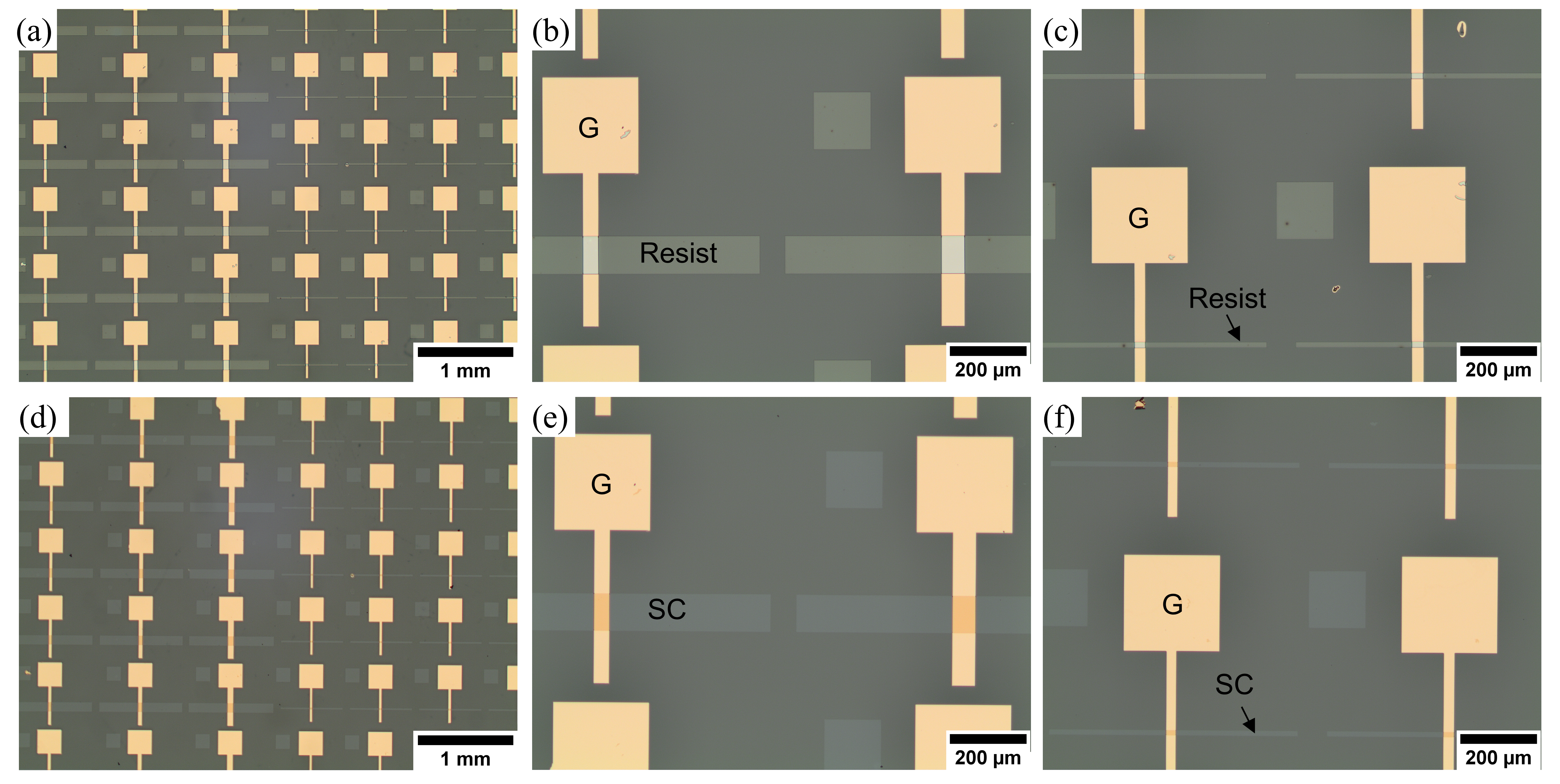


**Figure S5.** Optical microcopy images of the ROP patterning of ZnO layer. Top row from (a) to (c): polymer resist printed on top of ZnO layer and etched for 15 s. Bottom row from (d) to (f): same areas as above after resist removal with methanol.


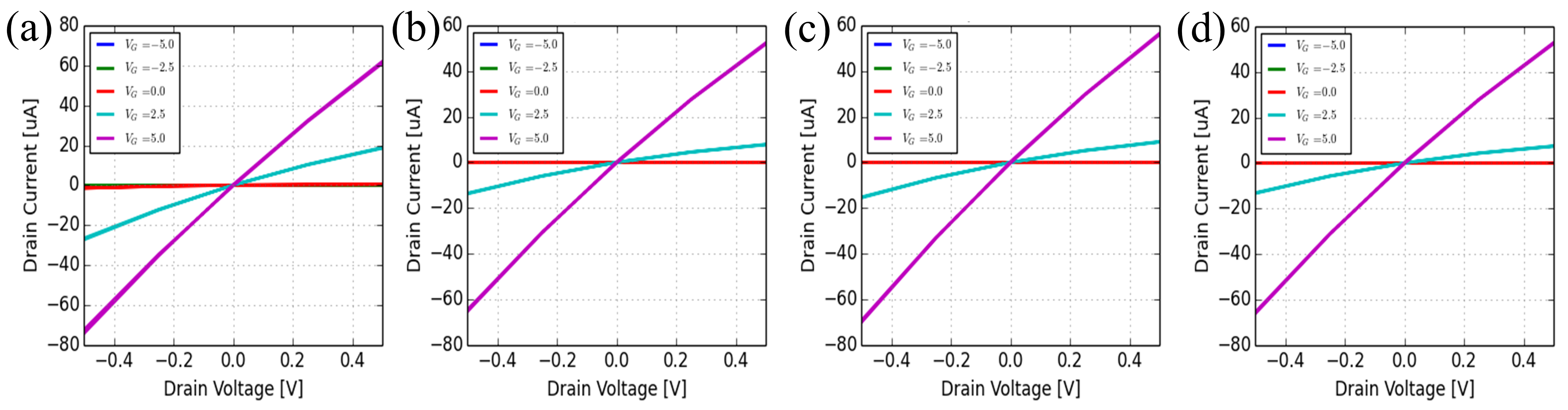


**Figure S6.** Output curves of a typical ZnO TFT with Ti/Au S/D at low V_d_ range (W = 200 µm / L = 20 µm) measured (a) 0 days, (b) 12 days, (c) 28 days and (d) 59 days after device fabrication.


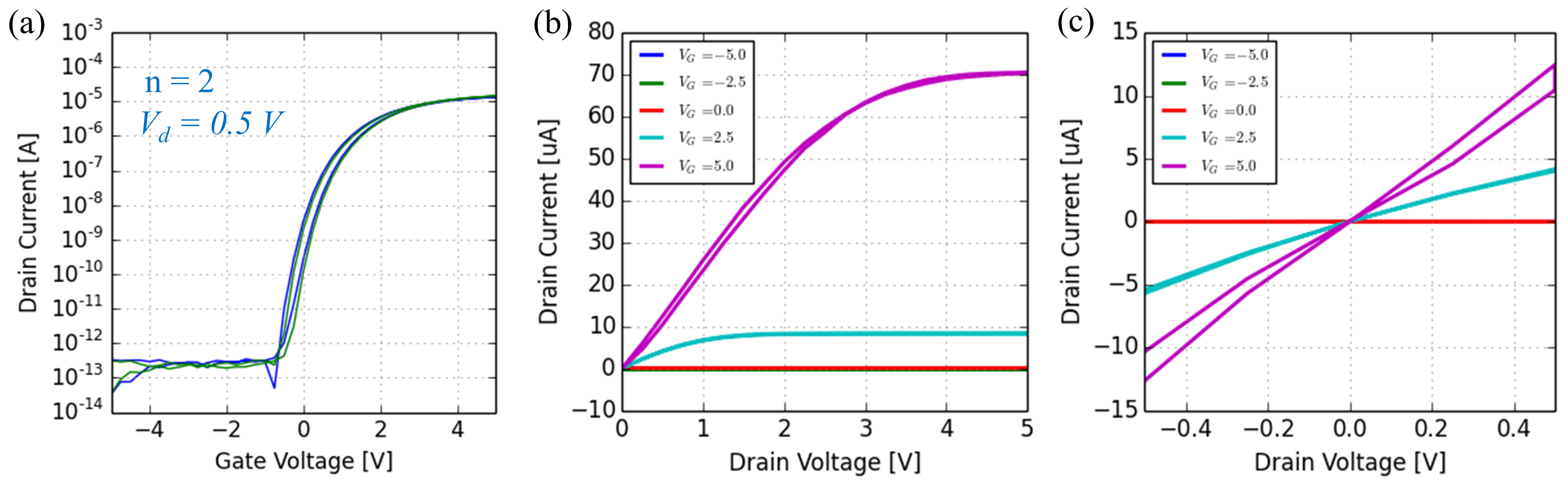


**Figure S7.** (a) Transfer curves of ALD-grown ROP-patterned ZnO TFTs with Al S/D (W = 200 µm / L = 20 µm). Output curve of a typical ZnO TFT with (b) V_d_ = 0 to 5 V and (c) V_d_ = -0.5 to 0.5 V (at low Vd range).

**Table S3.** Detailed electrical characterization parameters calculated based on data from Figure S7 (a).

| **ZnO TFTs with Aluminum (Al) S/D** |  |
| --- | --- |
| **Number of TFTs (n)** | n = 2 (n_total_ = 3) |
| **W/L (µm)** | 200/20 |
| **µ_FE_ (cm^2^(Vs)^-1^)** | 3.11 ± 0.09 |
| **V_on_ (V)** | -0.98 ± 0.07 |
| **V_t_ (V)** | 2.12 ± 0.12 |
| **V_hyst_ (V)** | 0.265 ± 0.005 |
| **I_on_/I_off_** | 5 × 10^7^ ± 3 × 10^6^ |
| **SS (V/decade)** | 0.17 ± 0.01 |

**Table S4.** Detailed electrical characterization parameters of ALD-grown ROP-patterned ZnO TFTs with Ti/Au S/D having different W/L ratios measured after 59 days.

| **ZnO TFTs with Ti/Au S/D - measured after 59 days** | | | | |
| --- | --- | --- | --- | --- |
| **n** | n = 1 (n_total_ = 3) | n = 2 (n_total_ = 3) | n = 3 (n_total_ = 3) | n = 2 (n_total_ = 3) |
| **W/L (µm)** | 200/5 | 200/10 | 200/20 | 200/40 |
| **µ_FE_ (cm^2^(Vs)^-1^)** | 13.4 | 15.7 ± 0.5 | 16.6 ± 0.4 | 17.1 ± 0.2 |
| **V_on_ (V)** | -0.671 | -0.52 ± 0.03 | -0.49 ± 0.02 | -0.451 |
| **V_t_ (V)** | 0.302 | 0.56 ± 0.03 | 0.71 ± 0.02 | 0.817 ± 0.002 |
| **V_hyst_ (V)** | 0.12 | 0.11 ± 0.02 | 0.13 ± 0.04 | 0.155 ± 0.005 |
| **I_on_/I_off_** | 9.73 × 10^8^ | 6 × 10^8^ ± 3 × 10^7^ | 2.8 × 10^8^ ± 1.1 × 10^7^ | 1.6 × 10^8^ ± 1.1 × 10^7^ |
| **SS (V/decade)** | 0.08 | 0.12 | 0.14 | 0.12 |

**Table S5.** Summary of R_S/D_, r_ch_ and adjusted R^2^ based on Figure 6 (a).

| **V_g_ (V)** | **R_S/D_ (Ω)** | **Channel resistivity r_ch_ (Ω/µm)** | **Adjusted R^2^** |
| --- | --- | --- | --- |
| **5** | 260 ± 90 | 455 ± 4 | 0.9998 |
| **4.5** | 230 ± 130 | 594 ± 6 | 0.99974 |
| **4** | 100 ± 300 | 815 ± 9 | 0.99964 |
| **3.5** | -200 ± 400 | 1190 ± 20 | 0.99949 |
| **3** | -800 ± 700 | 1900 ± 30 | 0.99928 |
| **2.5** | -2900 ± 1500 | 3440 ± 70 | 0.99899 |


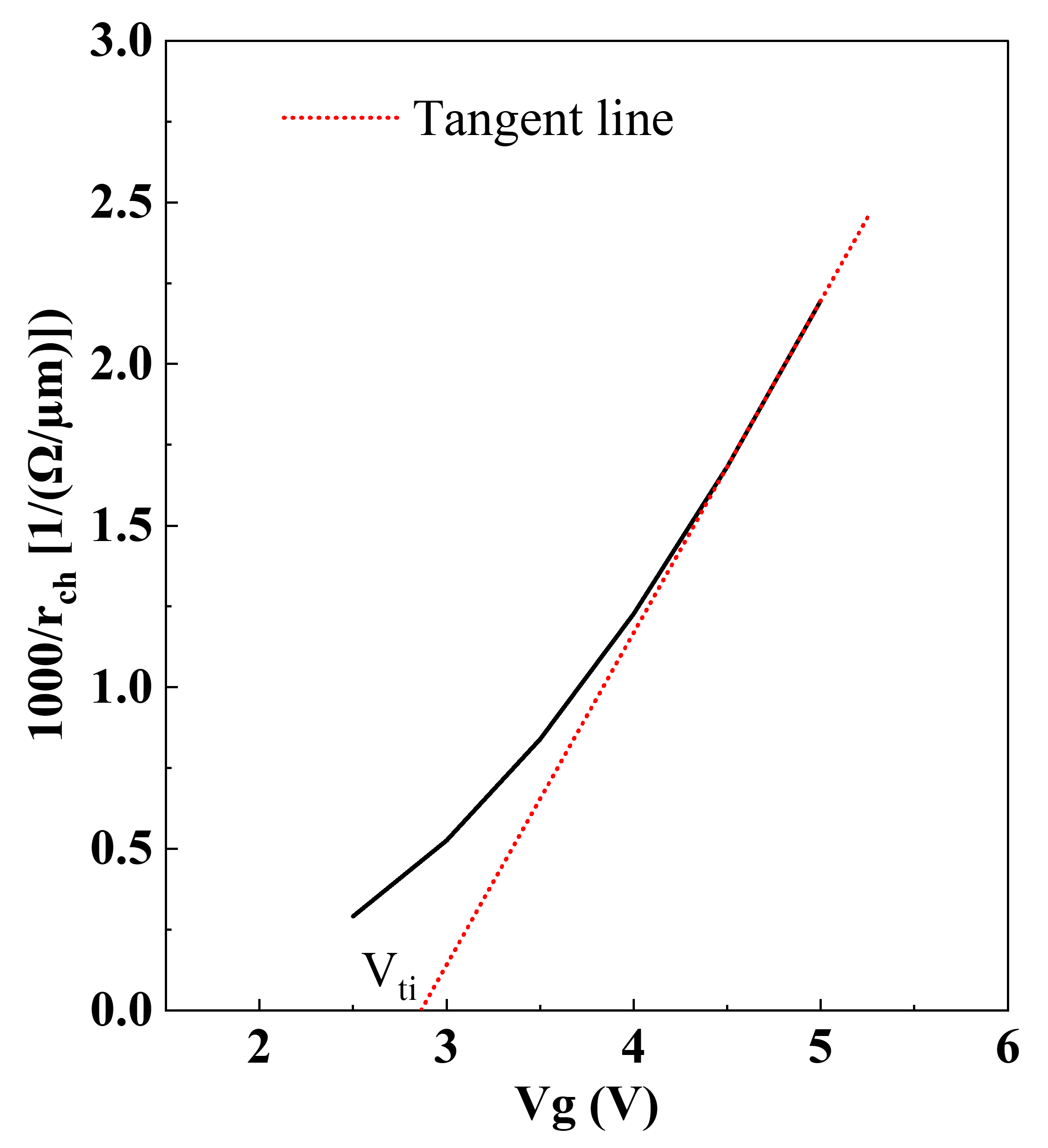


**Figure S8.** Plot of reciprocal of r_ch_ as a function of V_g_ for calculating μ_in_.


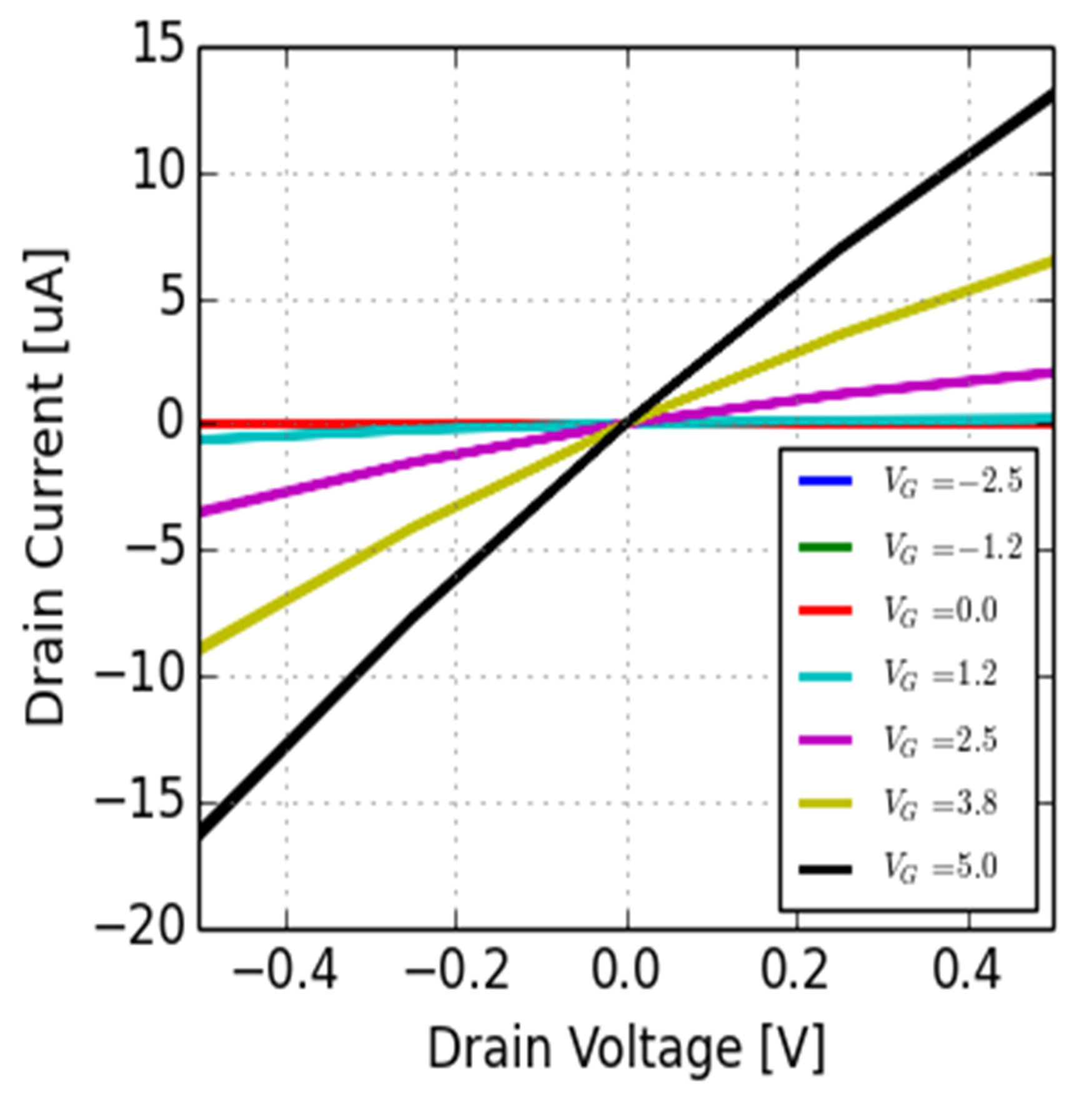


**Figure S9.** Output curve of a typical ZnO TFT with Ti/Au S/D at low V_d_ range (W = 20 µm / L = 7.5 µm).

REFERENCES

(1) Saha, J. K.; Bukke, R. N.; Mude, N. N.; Jang, J. Significant Improvement of Spray Pyrolyzed ZnO Thin Film by Precursor Optimization for High Mobility Thin Film Transistors. *Sci. Rep.* **2020**, *10* (1), 1–11. https://doi.org/10.1038/s41598-020-65938-6.

(2) Saha, J. K.; Ali, A.; Bukke, R. N.; Kim, Y. G.; Islam, M. M.; Jang, J. Performance Improvement for Spray-Coated ZnO TFT by F Doping with Spray-Coated Zr-Al-O Gate Insulator. *IEEE Trans. Electron Devices* **2021**, *68* (3), 1063–1069. https://doi.org/10.1109/TED.2021.3051918.

(3) Afouxenidis, D.; Mazzocco, R.; Vourlias, G.; Livesley, P. J.; Krier, A.; Milne, W. I.; Kolosov, O.; Adamopoulos, G. ZnO-Based Thin Film Transistors Employing Aluminum Titanate Gate Dielectrics Deposited by Spray Pyrolysis at Ambient Air. *ACS Appl. Mater. Interfaces* **2015**, *7* (13), 7334–7341. https://doi.org/10.1021/acsami.5b00561.

(4) Jeong, J. H.; Shin, J. S.; Ma, J. H.; Kang, S. J.; Kang, S. J. Highly Enhanced Visible Light Photodetection Properties of a ZnO Phototransistor via an Additional Solution Processed Thin Al2O3 Layer. *J. Mater. Chem. C* **2022**, *10* (39), 14770–14777. https://doi.org/10.1039/d2tc02500k.

(5) Jiang, L.; Li, J.; Huang, K.; Li, S.; Wang, Q.; Sun, Z.; Mei, T.; Wang, J.; Zhang, L.; Wang, N.; Wang, X. Low-Temperature and Solution-Processable Zinc Oxide Transistors for Transparent Electronics. *ACS Omega* **2017**, *2* (12), 8990–8996. https://doi.org/10.1021/acsomega.7b01420.

(6) Lee, J.; Jae, M.; Hassan, S. Z.; Chung, D. S. Sublimation-Doping with Super Bases for High-Performance Solution-Processed Heterojunction Oxide Thin Film Transistors. *Mater. Horizons* **2021**, *8* (11), 3105–3112. https://doi.org/10.1039/d1mh00929j.

(7) Kumar, M.; Jeong, H.; Lee, D. Solution-Processed High-Mobility ZnO Thin Film Transistors Based on Multiple-Stacked Channel Layer Doped with Hf and Mg. *Superlattices Microstruct.* **2018**, *120* (May), 395–401. https://doi.org/10.1016/j.spmi.2018.06.003.

(8) Yu, S. H.; Kim, B. J.; Kang, M. S.; Kim, S. H.; Han, J. H.; Lee, J. Y.; Cho, J. H. In/Ga-Free, Inkjet-Printed Charge Transfer Doping for Solution-Processed ZnO. *ACS Appl. Mater. Interfaces* **2013**, *5* (19), 9765–9769. https://doi.org/10.1021/am402919f.

(9) Hong, K.; Kim, Y. H.; Kim, S. H.; Xie, W.; Xu, W. D.; Kim, C. H.; Frisbie, C. D. Aerosol Jet Printed, Sub-2 v Complementary Circuits Constructed from P-and N-Type Electrolyte Gated Transistors. *Adv. Mater.* **2014**, *26* (41), 7032–7037. https://doi.org/10.1002/adma.201401330.

(10) Wang, C. J.; You, H. C.; Ou, J. H.; Chu, Y. Y.; Ko, F. H. Ultraviolet Photodetecting and Plasmon-to-Electric Conversion of Controlled Inkjet-Printing Thin-Film Transistors. *Nanomaterials* **2020**, *10* (3). https://doi.org/10.3390/nano10030458.

(11) Cunha, I.; Martins, J.; Bahubalindruni, P. G.; Carvalho, J. T.; Rodrigues, J.; Rubin, S.; Fortunato, E.; Martins, R.; Pereira, L. Handwritten and Sustainable Electronic Logic Circuits with Fully Printed Paper Transistors. *Adv. Mater. Technol.* **2021**, *6* (12), 1–12. https://doi.org/10.1002/admt.202100633.

(12) Jeong, Y. J.; Bae, J.; Nam, S.; Lim, S.; Jang, J.; Kim, S. H.; Park, C. E. Directly Drawn ZnO Semiconductors and MWCNT/PSS Electrodes via Electrohydrodynamic Jet Printing for Use in Thin-Film Transistors: The Ideal Combination for Reliable Device Performances. *Org. Electron.* **2016**, *39*, 272–278. https://doi.org/10.1016/j.orgel.2016.10.020.

(13) Pan, Z.; Peng, W.; Li, F.; He, Y. Carrier Concentration-Dependent Piezotronic and Piezo-Phototronic Effects in ZnO Thin-Film Transistor. *Nano Energy* **2018**, *49* (May), 529–537. https://doi.org/10.1016/j.nanoen.2018.05.005.

(14) Dong, J.; Han, D.; Li, H.; Yu, W.; Zhang, S.; Zhang, X.; Wang, Y. Effect of Al Doping on Performance of ZnO Thin Film Transistors. *Appl. Surf. Sci.* **2018**, *433*, 836–839. https://doi.org/10.1016/j.apsusc.2017.10.071.

(15) Alghamdi, W. S.; Fakieh, A.; Faber, H.; Lin, Y. H.; Lin, W. Z.; Lu, P. Y.; Liu, C. H.; Salama, K. N.; Anthopoulos, T. D. Impact of Layer Thickness on the Operating Characteristics of In2O3/ZnO Heterojunction Thin-Film Transistors. *Appl. Phys. Lett.* **2022**, *121* (23). https://doi.org/10.1063/5.0126935.

(16) Nogueira, G. L.; Kumar, D.; Zhang, S.; Alves, N.; Kettle, J. Zero Waste and Biodegradable Zinc Oxide Thin-Film Transistors for UV Sensors and Logic Circuits. *IEEE Trans. Electron Devices* **2023**, *70* (4), 1702–1709. https://doi.org/10.1109/TED.2023.3249126.

(17) Yang, J.; Bahrami, A.; Ding, X.; Lehmann, S.; Kruse, N.; He, S.; Wang, B.; Hantusch, M.; Nielsch, K. Characteristics of ALD-ZnO Thin Film Transistor Using H2O and H2O2 as Oxygen Sources. *Adv. Mater. Interfaces* **2022**, *9* (15), 2101953. https://doi.org/10.1002/admi.202101953.

(18) Wang, W.; Li, K.; Lan, J.; Shen, M.; Wang, Z.; Feng, X.; Yu, H.; Chen, K.; Li, J.; Zhou, F.; Lin, L.; Zhang, P.; Li, Y. CMOS Backend-of-Line Compatible Memory Array and Logic Circuitries Enabled by High Performance Atomic Layer Deposited ZnO Thin-Film Transistor. *Nat. Commun.* **2023**, *14* (1), 1–11. https://doi.org/10.1038/s41467-023-41868-5.

(19) Zhao, W.; Zhang, N.; Yao, C.; Zhang, J.; Huang, T.; Liu, Y.; Dong, S.; Ye, Z.; Luo, J. Enhanced Stability Performance of Transparent Ozone ALD ZnO Thin-Film Transistors with SiAlOXDielectric. *IEEE Trans. Electron Devices* **2023**, *70* (2), 556–562. https://doi.org/10.1109/TED.2022.3231817.

(20) Lin, Z.; Wang, Z.; Zhao, J.; Li, X.; Si, M. A Low-Leakage Zinc Oxide Transistor by Atomic Layer Deposition. *IEEE Electron Device Lett.* **2023**, *44* (3), 536–539. https://doi.org/10.1109/LED.2022.3233943.

(21) Li, S.; Chen, X.; Liu, L.; Zeng, Z.; Chang, S.; Wang, H.; Wu, H.; Long, S.; Liu, C. Micron Channel Length ZnO Thin Film Transistors Using Bilayer Electrodes. *J. Colloid Interface Sci.* **2022**, *622*, 769–779. https://doi.org/10.1016/j.jcis.2022.04.016.

(22) Chen, X.; Wan, J.; Gao, J.; Wu, H.; Liu, C. Enhanced Negative Bias Illumination Stability of ZnO Thin Film Transistors by Using a Two-Step Oxidation Method. *IEEE Trans. Electron Devices* **2022**, *69* (5), 2404–2408. https://doi.org/10.1109/TED.2022.3159284.

(23) Zhao, W.; Zhang, N.; Zhang, X.; Yao, C.; Zhang, J.; Dong, S.; Liu, Y.; Ye, Z.; Luo, J. Improvement in Instability of Transparent ALD ZnO TFTs Under Negative Bias Illumination Stress With SiO/AlO Bilayer Dielectric. *IEEE J. Electron Devices Soc.* **2022**, *10* (September), 927–932. https://doi.org/10.1109/JEDS.2022.3212477.

(24) Levy, D. H.; Ellinger, C. R.; Nelson, S. F. Metal-Oxide Thin-Film Transistors Patterned by Printing. *Appl. Phys. Lett.* **2013**, *103* (4). https://doi.org/10.1063/1.4816322.

(25) Liu, Y. C.; Hsieh, J. H.; Tung, S. K. Extraction of Optical Constants of Zinc Oxide Thin Films by Ellipsometry with Various Models. *Thin Solid Films* **2006**, *510* (1–2), 32–38. https://doi.org/10.1016/j.tsf.2005.10.089.
